# Supplementary material for: Burden, determinants, consequences and care of multimorbidity in rural and urbanising Telangana, India: protocol for a mixed-methods study within the APCAPS cohort
Source: BMJ Open. 2023 Nov 27;13(11):e073897. doi: 10.1136/bmjopen-2023-073897 (PMC10685937; doi:10.1136/bmjopen-2023-073897)
Supplement: Author's manuscript [file bmjopen-2023-073897.draft_revisions.pdf]

BMJ Open is committed to open peer review. As part of this commitment we make the peer review history of every article we publish publicly available.

When an article is published we post the peer reviewers' comments and the authors' responses online. We also post the versions of the paper that were used during peer review. These are the versions that the peer review comments apply to.

The versions of the paper that follow are the versions that were submitted during the peer review process. They are not the versions of record or the final published versions. They should not be cited or distributed as the published version of this manuscript.

BMJ Open is an open access journal and the full, final, typeset and author-corrected version of record of the manuscript is available on our site with no access controls, subscription charges or pay-per-view fees (http://bmjopen.bmj.com).

If you have any questions on BMJ Open's open peer review process please email info.bmjopen@bmj.com

BMJ Open

Burden, determinants, consequences, and care of multimorbidity in rural and urbanising Telangana, India: Protocol for a mixed-methods study within the APCAPS Cohort

Journal:	*BMJ Open*
Manuscript ID	bmjopen-2023-073897
Article Type:	Protocol
Date Submitted by the Author:	22-Mar-2023

Complete List of Authors:	Lieber, Judith; London School of Hygiene and Tropical Medicine Faculty of Epidemiology and Public Health,<br>Banjara, Santosh Kumar; National Institute of Nutrition<br>Mallinson, Poppy; London School of Hygiene and Tropical Medicine Faculty of Epidemiology and Population Health, Department of Non-communicable Disease Epidemiology<br>Mahajan, Hemant; National Institute of Nutrition<br>Bhogadi, Santhi; Public Health Foundation of India<br>Addanki, Srivalli; Public Health Foundation of India<br>Birk, Nick; London School of Hygiene and Tropical Medicine Faculty of Epidemiology and Public Health, Department of Non-communicable Disease Epidemiology<br>Song, Wenbo; London School of Hygiene and Tropical Medicine Faculty of Epidemiology and Public Health; Nagasaki University<br>Shah, Anoop ; London School of Hygiene & Tropical Medicine, Centre for Global Chronic Conditions, Faculty of Epidemiology and Population Health, Department of Non-communicable Disease Epidemiology,<br>Kurmi, Om ; Coventry University<br>Iyer, Gowri; Indian Institute of Public Health Hyderabad<br>Sureshkumar, K; Public Health Foundation of India, SACDIR; London School of Hygiene & Tropical Medicine, International Center for Evidence in Disability<br>Kishore Galla, Raghu; Enact Health Private Limited<br>Sadanand, Shilpa; Indian Institute of Public Health Hyderabad<br>Dasi, Teena; National Institute of Nutrition<br>Kulkarni, Bharati; Indian Council of Medical Research; National Institute of Nutrition<br>Kinra, Sanjay; London School of Hygiene and Tropical Medicine Faculty of Epidemiology and Public Health

Keywords:	EPIDEMIOLOGIC STUDIES, Aging, Cardiac Epidemiology < CARDIOLOGY, Dementia, General diabetes < DIABETES & ENDOCRINOLOGY, Risk Factors

Burden, determinants, consequences, and care of multimorbidity in rural and urbanising Telangana, India: Protocol for a mixed-methods study within the APCAPS Cohort

Judith Lieber[1]*, Santosh Kumar Banjara[2]*, Poppy AC Mallinson[1], Hemant Mahajan[2], Santhi Bhogadi[3], Srivalli Addanki[3], Nick Birk[1], Wenbo Song[1], Anoop Shah[1], Om Kurmi[4], Gowri Iyer[5], Sureshkumar Kamalakannan[1,5], Raghu Kishore Galla[6], Shilpa Sadanand[5], Teena Dasi[2], Bharati Kulkarni[2,7], Sanjay Kinra[1]

* Contributed equally.

1 – London School of Hygiene and Tropical Medicine, London, WC1E 7HT, UK

2 – National Institute of Nutrition, Indian Council of Medical Research, Hyderabad 50007, India

3 – Indian Instiitute of Public Health, Bengaluru, 560070, India

4 – Coventry University, Coventry, CV1 5FB, UK

5 - Indian Institute of Public Health, Hyderabad 500033, India

6 – Enact Health Private Limited, Hyderabad, 500018, India

7 – Indian Council of Medical Research, New Delhi, 110029, India

Contact details:

Judith.lieber1@lshtm.ac.uk; drsantoshkumar999@gmail.com; poppy.mallinson@lshtm.ac.uk; hemant.mahajan.84@gmail.com; kammilisanthi.mms@gmail.com; srivalli.addanki@gmail.com; Nick.Birk@lshtm.ac.uk; Wenbo.song@lshtm.ac.uk; Anoop.shah@lshtm.ac.uk; omkurmi@googlemail.com; gowri.iyer@iiphm.org; Suresh.Kumar@lshtm.ac.uk; raghukishoregalla@gmail.com; shilpa848@gmail.com; teena.dasi@icmr.gov.in; dr.bharatikulkarni@gmail.com; sanjay.kinra@lshtm.ac.uk

Corresponding author: Dr Judith Lieber; Judith.lieber1@lshtm.ac.uk; +44 (0)20 7927 2482; London School of Hygiene and Tropical Medicine, London, WC1E 7HT, UK.

Keywords

Risk factors, epidemiologic studies, aging, cardiac epidemiology, dementia, diabetes.

Abstract

Introduction: The epidemiological and demographic transitions are leading to a rising burden of multimorbidity (co-occurrence of two or more chronic conditions) worldwide. Evidence on the burden, determinants, consequences, and care of multimorbidity in rural and urbanising India is limited, partly due to a lack of longitudinal and objectively measured data on chronic health conditions. We will conduct a mixed-methods study nested in the prospective Andhra Pradesh Children and Parents' Study (APCAPS) cohort to develop a data resource for understanding the epidemiology of multimorbidity in rural and urbanising India and developing interventions to improve the prevention and care of multimorbidity.

Methods and analysis: We aim to recruit 2,100 APCAPS cohort members aged 45-plus who have clinical and lifestyle data collected during a previous cohort follow-up (2010-12). We will screen for locally prevalent non-communicable, infectious, and mental health conditions, alongside cognitive impairments, disabilities, and frailty, using a combination of self-reported clinical diagnosis,

For peer review only - http://bmjopen.bmj.com/site/about/guidelines.xhtml

symptom-based questionnaires, physical examinations, and biochemical assays. We will conduct in-depth interviews with people with varying multimorbidity clusters, their informal carers, and local healthcare providers. De-identified data will be made available to external researchers.

Ethics and dissemination: The study has received approval from the ethics committees of the National Institute of Nutrition and Indian Institute of Public Health Hyderabad, India, and the London School of Hygiene and Tropical Medicine, UK. Meta-data and data collection instruments will be published on the APCAPS website alongside details of existing APCAPS data and the data access process (www.apcaps.lshtm.ac.uk).

Strengths and limitations of the study

- Open-access multimorbidity resource of 2,100 adults aged 45-plus participating in the community-based APCAPS cohort in rural and urbanising Telangana, India, with scope to conduct longitudinal analyses to examine biological, behavioural, sociodemographic, and environmental risk factors for multimorbidity, multimorbidity progression and consequences.
- A broad range of locally prevalent chronic conditions screened using objective measures to estimate the burden of multimorbidity, overcoming the limitations of self-reporting.
- A qualitative approach to explore the lived experience of people with multimorbidity and their informal carers and identify current gaps in care to guide intervention and policy development.
- Statistical power may be insufficient to examine some less prevalent clusters of multimorbidity.
- The study population is based in a defined geographical area that does not represent the entire population of India and may not capture multimorbidity clusters specific to other Indian settings.

Word count: 3917 (not including tables, references, abstract, headings, figures).

For peer review only

Introduction

Multimorbidity, defined as the co-occurrence of two or more conditions of long-duration ('chronic') in an individual, is rising globally (1). Multimorbidity is associated with poor quality of life, disability, mortality, and healthcare use, which significantly strain health systems (1). In rural India, where 65% of the Indian population reside, socioeconomic developments and globalisation are introducing new risk factors for chronic conditions (e.g., air pollution) alongside prevailing poverty-related risks (e.g., undernutrition). The rising prevalence of non-communicable diseases alongside an ongoing burden of infectious disease is leading to a significant rise in multimorbidity (2,3). Although a system for primary care exists, the majority of outpatient health services are provided by the private sector (4). Lack of a robust system of primary care leaves people with multimorbidity highly vulnerable to catastrophic health expenditure and poor health and social outcomes (5,6). Evidence on the burden, determinants, consequences, and gaps in care of multimorbidity is required to improve the prevention and care of multimorbidity in rural and urbanising India and other settings undergoing similar trends (2).

Current evidence on the burden of multimorbidity in rural and urbanising India is limited. Incidence data are lacking, while prevalence estimates have fluctuated widely (ranging from 9 to 59%) owing to a frequent reliance on self-reported disease, which is known to grossly underestimate true disease prevalence in these settings (6–9). There is some evidence that multimorbidity clusters in India mirror those reported in high-income countries, specifically, clustering of cardio-respiratory (angina, asthma, chronic obstructive pulmonary disease (COPD)), metabolic (diabetes, obesity, hypertension) and mental-articular (arthritis and depression) conditions (10–14). However, multimorbidity clusters specific to rural and urbanising India may have not been identified as several locally prevalent conditions (e.g., anaemia) were not measured in prior studies (9–11,13,14).

Longitudinal studies from high-income countries have identified several risk factors (e.g., air pollution) associated with multiple chronic conditions (e.g., cardio-respiratory, cancers) and social determinants of multimorbidity (15,16). However, longitudinal evidence on the risk-factors for multimorbidity is critically lacking in India and other low- and middle-income countries (LMICs) (17,18). While a few longitudinal studies from India have investigated the risk factors of individual chronic conditions, the effect of these or other common risk factors in rural India (e.g., indoor use of biomass fuel) on multimorbidity has not been examined (7).

Studies in high-income countries have reported the challenges of caring for multimorbidity for the individual as well as their informal carers (e.g., impacted social relationships), and healthcare providers (e.g., lack of clinical guidance for treatment of multiple conditions) (19–21). Studies of individual conditions in India have highlighted long, complex and burdensome care pathways (22,23). However, evidence of the lived experience of multimorbidity for patients and their informal carers is lacking in India, particularly in rural areas. Further, few studies have explored the barriers to care of multimorbidity in India, focusing specifically on diabetes comorbidities (22,24).

Aim and objectives

We aim to develop a longitudinal data resource for examining the epidemiology of multimorbidity in rural and urbanising India and developing interventions to improve the prevention and care of multimorbidity. We envisage that this will also have relevance for other rural and urbanising settings at similar stages in their epidemiological transition. Our objectives are:

1. To estimate the prevalence and incidence of multimorbidity (defined as two or more chronic conditions) and common multimorbidity clusters in rural and urbanising Telangana, India.

For peer review only

2. To examine the biological, behavioural, sociodemographic, and environmental risk factors for, and consequences of, multimorbidity.
3. To explore the lived experience of individuals with multimorbidity and their informal carers.
4. To assess how care for multimorbidity is organised in rural and urbanising India and identify the barriers and enablers to high-quality care.

Methods and analysis

Study design

A mixed-methods study in the community-based Andhra Pradesh Children and Parents Study (APCAPS) cohort (25). The qualitative methods (objectives 3-4) will be embedded within the quantitative study (objectives 1-2), using the quantitative data as a sampling frame.

Study population and setting.

APCAPS is a prospective inter-generational cohort that has been incrementally built through long-term follow-up of a nutrition trial (1987-90) conducted in 29 villages (Ranga Reddy district) in the south Indian state of Telangana. The 29 villages of the APCAPS cohort are located 50-100 km from Hyderabad, the state capital, and have urbanised at varying rates, now presenting a mix of rural and urban risk factors. For children born during the original nutrition trial, chronic disease risk factor and outcome data has been collected repeatedly (2003-05 to 2010-12). In 2010-12, similar data was collected for their parents and siblings (N=6,972 total cohort). Figure 1 provides an overview of the longitudinal multimorbidity analyses feasible with the 2010-12 and 2022-23 data.

Inclusion criteria

Adults aged 45 years and above who participated in a prior APCAPS study residing in the study villages or surrounding area are eligible to participate. This group primarily comprises the parents' generation of the cohort plus older siblings of the trial children. While the target population are aged 45-plus, cohort members aged 18-44 are eligible to undergo a limited protocol if they wish to participate.

Exclusion criteria

None.

Quantitative data collection

Figure 1 here.

Quantitative sample

A cohort tracking exercise (April-August 2021) identified N=2,571 APCAPS members eligible to participate (58% women). We anticipate a final sample size of N=2,057 (expected mean age 63 and 56 years for men and women, respectively) based on an estimated 80% follow-up. The proposed sample size will allow us to estimate the 10-year incidence of any multimorbidity (defined as two or more chronic conditions), and incidence of specific common multimorbidity clusters, with a precision of 4% and 3% respectively (assuming a conservative N=700 new cases of multimorbidity and three equal-sized clusters) (10). For analysis of risk factors, we will have statistical power to detect a rate ratio of ~1.25 (assuming an incidence rate of 35/1000 person-years in the unexposed with a 30% risk factor prevalence and 80% power at a 5% significance level).

Quantitative data collection procedures

For peer review only

Following piloting, data collection commenced in February 2022 and will be completed towards the end of 2023. Data collection is occuring village-by-village, with clinics occurring in the study villages or at the central study office (ICMR-National Institute of Nutrition (NIN), Hyderabad), or the household when necessary, e.g., for people with mobility impairments. Questionnaires and physical examinations occur at the clinic, and participants are invited to return the following weekend to provide blood and urine samples. Questionnaire responses and results of most physical assessments are collected by tablet using the Open Data Kit (ODK) platform (26). Other data are extracted directly from the relevant instruments.

Quantitative protocol

The full protocol consists of questionnaires, a physical examination, and collection of urine and fasting blood samples. The screened conditions were selected based on their reported prevalence in India and their importance to the local community (27–30). We are screening for chronic diseases with a combination of self-reported diagnosis by a healthcare professional, symptom-based questionnaires, physical examinations, and biochemical assays, in order to overcome the socioeconomic bias observed when defining conditions by prior clinical diagnosis in rural India (8) (table 1). The questionnaires cover sociodemographics, behavioural risk factors, self-reported clinical diagnosis of chronic conditions, treatment details, symptom-based screening tools, and impact measures (e.g., quality of life). We limit questions about prior clinical diagnosis to conditions with a higher likelihood of diagnosis in the study population, based on assessment of existing and pilot APCAPS data and discussions with local healthcare providers. The protocol was designed to maximise the scope for longitudinal analyses and to measure key chronic conditions and impact outcomes (31).

The physical assessment includes a detailed examination of cardiovascular physiology, anthropometry and physical functioning assessments, and assessment of diabetes complications (neuropathy and ulceration) for participants reporting a prior diagnosis of diabetes or screening positive for pre-diabetes/diabetes. Individuals with limb or spine deformities are excluded from the anthropometry protocol, and individuals with mobility impairments are excluded from the 6-meter timed walk if they/the fieldworker deem it unsafe. Pregnant women and individuals with a prior diagnosis of diabetes are not requested to fast overnight for blood sampling but are eligible to provide a sample. To limit participant burden given the extensive protocol, screening instruments were chosen based on validity in the study population and time taken to complete. As this is an ongoing cohort, we plan to prospectively collect mortality data, including cause of death.

Table 1: Screened health conditions and states of poor health, by type of screening measure

Health condition/state	Screening measure		
	Self-reported clinical diagnosis	Symptom-based questionnaire	Physical examination/ biochemical assay
Hypertension	✓		✓
Left ventricular dysfunction/Heart failure*	✓		✓
Coronary heart disease*	✓	✓	
Atrial fibrillation*	✓		✓
Atherosclerosis			✓
Stroke	✓	✓	

For peer review only

Underweight/obesity			✓
Diabetes	✓		✓
Diabetic ulceration	✓		✓
Diabetic neuropathy	✓	✓	✓
Peripheral arterial disease	✓	✓	✓
Retinopathy	✓		✓
Cataracts	✓	✓	
Chronic obstructive pulmonary disease		✓	
Asthma	✓	✓	
Sarcopenia			✓
Arthritis	✓	✓	
Anaemia			✓
Depression		✓	
Anxiety		✓	
Alcohol Use Disorder		✓	
Dementia		✓	
Tuberculosis	✓	✓	
COVID-19	✓	✓	
Thyroid dysfunction	✓	✓	✓
Chronic liver disease	✓		✓
Chronic kidney disease	✓		✓
Peptic ulcer	✓		
Gastro-oesophageal reflux disease	✓	✓	
Cancer	✓		
Poor oral health		✓	
Chronic pain		✓	
Chronic stress		✓	
Frailty		✓	✓
Disability		✓	✓
Vision impairment	✓		✓
Hearing impairment	✓		✓
*Question on prior clinical diagnosis refers to 'heart disease' but does not categorise by condition.			

For peer review only

Validity of quantitative protocol

Hypertension is assessed by seated brachial blood pressure (three readings) with validated oscillometric devices (32–34) (table 2). Left ventricular dysfunction is assessed by 2D-Echocardiography performed by an experienced echo technician following a standardised protocol (35), coronary heart disease symptoms with the validated WHO Rose questionnaire (36), atrial fibrillation with 12-lead ECG, and atherosclerosis with carotid-intima media thickness (CIMT). A stroke is measured with a symptom-based questionnaire developed and commonly used to estimate the community-based prevalence in India (37). Underweight and obesity are assessed with height, weight, and hip and waist circumferences to estimate body mass index (BMI) and waist-to-hip ratio, and bioelectrical impedance to estimate body fat percentage, with all measures taken twice and using the same standardised protocols as earlier APCAPS surveys (25).

Diabetes is screened by glycated haemoglobin (HbA1c) and Fasting Plasma Glucose, both gold-standard diagnostic tests (38). Participants with self-reported diabetes and/or who screen positive for pre-diabetes or diabetes (based on Fasting Plasma Glucose) are undergoing a diabetic neuropathy examination and assessment for diabetic ulceration. We do not predict advanced neuropathy in the study population due to their relatively young age (average 59 years). As such, we are using the validated modified Toronto Clinical Neuropathy Score (mTCNS) which aims to detect early dysfunction (39). Diabetic foot ulcerations are assessed with infrared thermal imaging, a validated measure (40). In all participants, peripheral arterial disease (PAD) is assessed by toe-brachial index (TBI) which has better sensitivity in people with diabetes (in whom we predict most cases of PAD) versus the more commonly used ankle-brachial index (41). Retinal images are being taken to screen for retinopathy, with two fields (centred on the optic disc and the macula) to improve validity (42). We are using a non-mydriatic camera without pupil dilation to maximise participant comfort. Cataracts are assessed with a symptom-based tool previously used in a nationwide survey in India (43).

To reduce risk of COVID-19 transmission, chronic respiratory conditions (COPD and asthma) are assessed with validated symptom-based questionnaires (44,45) instead of spirometry (used in prior APCAPS surveys (46)). Sarcopenia is defined by muscle strength, physical performance, and appendicular skeletal muscle mass (47). Muscle strength is measured by grip strength using the same instrument and protocol as the 2010-12 APCAPS survey as grip strength estimates are highly sensitive to instrument and protocol used (25,48). In line with the Asian Working Group for Sarcopenia's recommendations, physical functioning is being measured with a 6-meter timed walk at normal speed with a dynamic start, and appendicular skeletal muscle mass with bio-impedance analysis (47). Arthritis is screened with a cross-culturally valid symptom-based questionnaire (49). Anaemia is assessed by haemoglobin concentration of fasting venous blood with an auto-analyzer on the day of sample collection (a gold-standard diagnostic test (50)). Mental health conditions (depression, anxiety, and alcohol-use disorder) and dementia are being screened with widely used questionnaires validated for Indian populations (51–53). Due to the relatively low expected prevalence of tuberculosis (TB) in the study population, and high resource requirements and participant burden for diagnostic testing, we screen for TB with a bespoke symptom-based questionnaire. The questions are based on the Government of India's definition of presumptive TB and WHO guidance for TB surveys to allow within- and across-country prevalence comparisons respectively (54–56). COVID-19 history and post COVID-19 condition (Long COVID) are assessed with a combination of self-report and symptom-based questions devised for the study based on WHO case definitions (57,58). As we could not identify a validated tool that was sufficiently brief, thyroid dysfunction is assessed with questions developed for the study based on Zulewski's list of symptoms of hypothyroidism (59) and fieldworker's visual assessment of goitre (bespoke protocol based on the WHO case definition of goitre (60)). Liver disease is assessed with a 2D ultrasound to detect liver fibrosis and fatty liver combined with fasting blood biomarkers (table 3). Chronic kidney disease is assessed by estimated Glomerular Filtration Rate (eGFR) based on serum creatinine, weight, age and sex using appropriate equations, and urine albumin to creatinine ratio. Gastro-oesophageal reflux disease (GORD) is assessed with a symptom-based questionnaire developed for Indian populations (61).

As recommended by a recent working group of experts in multimorbidity (1), we are measuring additional states of poor health (poor oral health, chronic pain, frailty, chronic stress), as well as disability. Oral health is assessed with a symptom-based questionnaire of a nationwide survey in India (62). We define chronic pain as persistent or recurring pain lasting more than 3-months (in line with ICD-11), assessed with a questionnaire from the UK Biobank pain survey (as we

For peer review only

could not identify a sufficiently brief questionnaire validated in India (63,64)). We conceptualise frailty with the widely used Fried frailty phenotype (65): slowness (measured with the 6-m timed walk), physical activity (validated APCAPS physical activity questionnaire (66)), weakness (grip strength), weight loss (question devised for the study (TB symptom questionnaire)), and exhaustion (question in the depression tool, PHQ-9). The short (4-item) version of the cross-culturally validated Perceived Stress Scale is used to assess chronic psychological stress to minimise participant burden (67). As recommended by prior studies in rural India, disability is measured with a validated functional limitation tool previously tested in India (68,69), as well as screening for impairments. Vision impairment (near and distant) is measured with a standard visual acuity test protocol using the Tumbling E LogMAR chart, which is appropriate for people with low literacy. Hearing impairment is assessed with a smartphone-based hearing threshold test using the validated Hearing Test (pro) application (e-audiological.pl) (70). HearingTest has demonstrated good validity at higher frequencies (71,72); presbycusis (characterised by hearing loss at high frequencies) is likely the most common cause of hearing loss in the study population (73). Information on deaths occurring in the cohort will be collected from community health (ASHA) workers, who routinely monitor mortality in the villages, on a 6-monthly prospective basis (project funding permitting). Close contacts of the deceased will be interviewed using the validated WHO verbal autopsy instrument (2022 version) to ascertain cause of death (74).

Table 2: Measures collected in planned study (2022-23), source of questionnaire/instrument and protocol, and availability in prior (2010-2012) APCAPS study.

●-collected with same instrument, ⊖-collected with different instrument, ○-not collected in 2010-12.

Measure	Source of questionnaire/Instrument and protocol	Availability in 2010-12
**Questionnaires**		
Sociodemographics	APCAPS 2010-12 survey	●
Food insecurity	Household Food Insecurity Access Scale (9-item) (75)	○
Family health history	APCAPS 2010-12 survey	●
Tobacco use	APCAPS 2010-12 survey	●
Alcohol use	Validated APCAPS food frequency questionnaire (76)	●
Diet	Validated APCAPS food frequency questionnaire (76)	●
Physical activity	Validated APCAPS physical activity questionnaire (66)	●
Sleep	APCAPS 2010-12 survey	●
Medical history	APCAPS 2010-12 survey, photos of medicines.	●
CHD symptoms	WHO Rose Questionnaire (36)	●
PAD symptoms	WHO Rose Questionnaire (36)	○
Stroke	Gourie-Devy et al., survey (37)	⊖
Cataracts	SAGE survey	○
Chronic Obstructive Pulmonary Disease	Lung Function Questionnaire (44)	⊖
Asthma	European Community Respiratory Health Survey (short) (77)	⊖
Arthritis	SAGE survey	○
Depression	Patient Health Questionnaire-9 (PHQ-9) (78)	●
Anxiety	Generalised Anxiety Disorder-7 (GAD-7) (79)	⊖
Alcohol Use Disorder	Alcohol Use Disorders Identification Test (80)	○
Dementia	Brief Community Screening Instrument for Dementia (53)	○
Tuberculosis	Developed for study (Government of India's case definition of presumptive TB plus night sweats (54,55))	⊖

COVID-19, post COVID-19 condition	Developed for study (WHO case definitions) (57,58)	○
Hypothyroidism	Developed for study (Zulewski case definition (59))	⊖
Gastro-Oesophageal Reflux Disease	Indian Society of Gastroenterology survey (61)	○
Oral health	LASI survey	○
Chronic pain	UK Biobank survey (63)	○
Chronic stress	Perceived Stress Scale (Short Version) (81)	○
Disability	Washington Group Short Set on Functioning (82)	⊖
Health-related quality of life	EuroQol 5-Dimension Health Questionnaire, 5-level (83)	●
Healthcare use	LASI survey	○
Medicine costs	LASI survey	○
Falls	Developed for study (Prevention of Falls Network Europe case definition (84))	○
Cause of death	2022 WHO Verbal Autopsy Instrument (74)	○
**Physical/ cognitive**		
Brachial blood pressure	Three readings taken in the right arm, sitting position (Omron HEM-7121 and Uscom BP+)	●
Heart structure and function	2D Echocardiogram (Philips CX-50 2D-ultrasound, S5-1 PureWave Cardiac Sector probe)	○
Arrhythmia	Resting ECG (12-lead 12-channel Cardiax ECG)	○
Carotid Intima-Media Thickness	Close to bifurcation (Philips CX-502D-ultrasound, linear probe, Philips QLAB software)	⊖
Arterial stiffness	Three readings taken in the right arm, sitting position (Uscom BP+ supra-systolic central blood pressure monitor)	⊖
Standing height	End of expiration to nearest 1mm (Seca Leicester stadiometer)	●
Weight	Single frequency bioelectrical impedance (BIA) scale (TANITA BC418 M57NA) or, if unable to undergo BIA, digital scales to the nearest 0.1kg (Seca 899)	●
Body composition	Single frequency BIA scale (TANITA BC418 M57NA), two measures taken wearing light clothes and bare feet.	●
Waist circumference	Narrowest point of the waist from the front (ADE non-stretch metallic tape)	●
Hip circumference	Maximum extension of buttock from the side (ADE non-stretch metallic tape)	●
Diabetic ulceration	Infrared images of both feet following 15-minute rest (CAT-S61 smartphone with FLIR thermal imaging camera)	○
Diabetes neuropathy	Sensory assessment of the foot and symptom-based questionnaire (mTCNS) (39)	○
Toe brachial index	Ratio of brachial and toe pressures (three measures), photoplethysmography method (Vicorder, Skidmore Medical), taken in a supine position after 5-minute rest.	○
Retinopathy	Retinal images centred on the optic nerve and macula taken in a darkened room without dilation (Bosch digital fundus camera (non-mydriatic))	○
Gait speed and characteristics	6-meter timed walk at normal speed with dynamic start and no deceleration with a waist-worn accelerometer (Actigraph)	○

For peer review only

Grip strength	Standing position, elbows at 20º angle, two measures in both hands (Lafayette Hand-held Dynamometer 78010)	●
Goitre	Visual assessment (WHO goitre case definition) (60)	○
Liver fibrosis	Ultrasound of right lobe with peri-portal vasculature visible (Philips CX-50 2D-ultrasound, C5-1 PureWave Convex probe)	○
Vision impairment	Tumbling E LogMAR chart (4m and 40cm distance) (Precision Vision)	○
Hearing impairment	Phone-based pure-tone audiometry (Hearing Test (pro) application (e-audiological.pl) (85), Sennheiser HD 458 BT headphones)	○
Retrieval fluency	Maximum animals named in 1-minute (animal naming test)	○
**Environment**		
Village-level urbanisation	Night-time light intensity remote sensing data (86)	●
Air pollution	PM2.5 and black carbon prediction models (87)	●
Built environment	Shops selling food, tobacco & alcohol, health services, educational services, advertisements, public transport and walkability, physical activity (surveyed and geolocated)	⊖*

APCAPS=Andhra Pradesh Children and Parents Study, CHD = Coronary Heart Disease, PAD = Peripheral Arterial Disease, PM2.5 = Particulate Matter 2.5, LASI = Longitudinal Aging Study in India, SAGE = Study on Global Ageing and Adult Health, WHS = World Health Survey, WHO = World Health Organisation. * Surveyed and geolocated in 2016, not updated in 2022-23.

Sample collection and processing

Participants are asked to fast overnight (at least 8 hours) and rest quietly for 10 minutes before venous blood samples (up to 20ml) are collected from their preferred arm by a trained phlebotomist (table 3). Blood are collected into appropriate vacutainers (2 plain, 1 EDTA, 1 Na-F EDTA, Becton Dickinson, New Jersey, United States) then centrifuged and aliquoted in situ within 1-4 hours. Samples are kept on ice until being transferred to -80°C freezers at the central laboratory, within six hours of collection. Participants are given a urine collection container the day before their scheduled clinic and instructed to collect a mid-stream urine sample from their first urination the next morning. Samples are kept on ice during the clinic and transferred to the central laboratory to be centrifuged (to remove cells/debris), aliquoted, and stored at -80°C that day.

Sample analysis

On the same day as sample collection, the following assays are being conducted at the central laboratory (ICMR-NIN, Hyderabad, India): Complete blood count using autoanalyzer (Horiba ABX Micros 60 OT, Japan) and plasma glucose using GOD-POD method (Randox laboratories India Pvt Ltd) (table 3). Appropriate 2-level or 3-level quality controls are in place as provided by the manufacturers. Other assays will be conducted in batches using frozen serum or plasma or urine samples.

Table 3: Biological samples collected in planned study (2022-23), protocol/assay, and availability in prior (2010-2012) APCAPS study.

●-collected with same instrument, ⊖-collected with different instrument, ○-not collected in 2010-12.

**Stored biological samples and assays**		
**Measure**	**Protocol/assay**	**Availability in 2010-12**

For peer review only

Blood	Venous blood (overnight fast), processed on the same day (plasma and serum aliquots) and stored at -80C	●
Urine	First urination of the day, mid-stream, processed on the same day and stored at -80C	○
Haemoglobin	Auto-analyser (ABX Micros 60 – HORIBA)	⊖
Fasting Blood Glucose	GOD-POD method (Randox laboratories, India Pvt Ltd)	●
HbA1c	To be confirmed	○
Serum lipids (HDL, TC, Trig)	To be confirmed	●
Liver function tests (ALT, AST, ALP Bilirubin)	To be confirmed	●
Serum creatinine	To be confirmed	●
Urine albumin to creatinine ratio	To be confirmed	○

HbA1c = glycated haemoglobin, HDL = high-density lipoprotein, TC = total cholesterol, Trig = triglycerides, ALT = alanine transaminase, AST = aspartate aminotransferase, ALP = alkaline phosphatase.

Quality of quantitative data

Fieldworkers were trained in consent procedures, ODK, survey interviewing, and the anthropometry examination (following the same protocols as prior APCAPS surveys) by the experienced APCAPS supervisory team (10-15 years working with the cohort). For measures not collected in prior studies (table 2), fieldworkers were either trained by coinvestigators (in their respective fields) or external experts. Developers of the mTCNS instrument and a neurophysician trained fieldworkers to conduct the neuropathy assessment (39). A clinical ophthalmologist trained fieldworkers to collect retinal images, provided quality control feedback on initial images collected and refresher training. Experienced technicians trained in 2D-Echo are undertaking the echocardiogram, liver scan, and CIMT. A team of experienced phlebotomists and biotechnicians are collecting, aliquoting and storing biological samples. Prior to data collection, the full protocol (questionnaires and physical examinations) was piloted in the community.

To maximise reliability, rigorous protocols have been developed, each measure is collected by the same 2-3 fieldworkers to minimise inter-rater variability, and instruments are calibrated at daily, weekly, monthly and/or yearly intervals based on relevant guidelines. The supervisory team observe, evaluate, and feedback on protocol implementation in the field using a quality control proforma. To minimise data errors, data collected through the ODK platform restricts implausible values, and other data is extracted directly from instruments when feasible. Data quality reports that describe missing data, variable distributions, and questionnaire completion times are assessed by the research team fortnightly. On an ongoing basis, quality of echocardiograms, ECG traces, and CIMT images are examined by an experienced cardiologist and the retinal images with an automated tool (88). The protocol is repeated in a random 5% of participants 2-4 weeks following initial data collection, intra-class correlation coefficients (ICCs) are estimated for key continuous variables (e.g., anthropometrics) as the study is ongoing, and refresher training conducted if ICCs indicate decreasing reliability.

To ensure correct data linkage, participants' personal details are used to link them to their unique APCAPS identifiers (ID) upon attending the clinic. Each participant has a folder with a QR

For peer review only

code linked to their APCAPS ID to track protocol completion, minimise missing data, link each dataset to the correct participant, and link incoming data to existing APCAPS data. Sample vials are labelled with QR codes linked to APCAPS IDs. During data cleaning, age and sex will be cross-checked across datasets, and discrepancies will be compared against raw data to ensure correct linkage.

Data analysis

Assessment of the burden of multimorbidity will include descriptive statistics of the prevalence and (where possible) 10-year cumulative incidence of health conditions assessed, both individually and combined into a multimorbidity score (count of conditions for each individual). Cluster analysis approaches will also be used to identify common clusters of multimorbidity to be taken forward in further analyses. Risk factors (e.g., occupation, air pollution) and consequences (e.g., health-related quality of life) of multimorbidity (overall score and specific prevalent clusters) will be assessed by multiple logistic or ordinal logistic regression modelling, adjusted for appropriate covariates and using multiple imputation to complete missing data values where necessary.

Qualitative data collection

Following quantitative data collection, we will use qualitative methods to explore the lived experience of individuals with multimorbidity and their informal carers, to understand how healthcare is organised for people with multimorbidity, and to identify the barriers and enablers to high-quality care of people with multimorbidity. Participants will include individuals with multiple diagnosed conditions, their informal carers (defined as someone who helps them with their health regularly), and local healthcare providers involved in the diagnosis and management of chronic conditions. This is guided by the Burden of Treatment Theory, which models how patients, their social networks, and the healthcare system interact to address the burden of treatment, a key issue in multimorbidity (19,89).

Qualitative sample

We will use a purposive sampling strategy aiming for maximum variation in clusters of diagnosed conditions. The quantitative data will be analysed using clustering approaches to identify the most common concordant, discordant, and complex multimorbidity clusters (defined by the number of conditions (four-plus) or complexity of treatment for individual conditions) (approximately N=15). Around half of the interviewees will be asked to refer an informal carer to participate. We will also aim for maximum variation in healthcare provider type, including doctors in local primary health centres and district hospitals, registered medical practitioners, and pharmacists (approximately N=15). Trancripts will be reviewed as data collection is ongoing, further interviews may be undertaken to explore issues being raised by participants.

Qualitative methods and analysis

We will undertake in-depth, one-to-one interviews with participants in the local language (Telugu), which will be audio-recorded. The Burden of Treatment Theory, plus existing evidence on diagnosis, management, and impact of chronic conditions in India, will guide development of the topic guides (89). Audio recordings will be translated and transcribed into English. We will use a reflective thematic analysis approach for analysis (90).

Qualitative data quality

Prior to data collection, topic guides will be piloted in the community to evaluate relevance, comprehension, and length. Interviews will be conducted by experienced qualitative interviewers.

For peer review only

To be sensitive to local gender norms and maximise participant comfort, interviewers of the same gender as participants will undertake the interviews in private locations (as feasible) of the participants' choosing. A subset of the early transcripts will be double-coded to clarify code definitions and initiate discussion and interpretations.

Patient and public involvement

The study design was based on insights from in-depth interviews and focus groups with community members and local health providers, who perceived a high burden of a range of cardiometabolic, mental health, and skeletal conditions, and sensory and mobility impairments in the population (27). The study design was discussed with and approved by village leaders.

Ethics

The study protocol and tools have been approved by the ethics committees of ICMR-NIN (CR/1/V/2023) and Indian Institute of Public Health Hyderabad (IIPHH/TRCIEC/189/2018), India, and the London School of Hygiene and Tropical Medicine (21771 /RR/19113), UK. Trained fieldworkers will verbally explain the study information sheet to potential participants; if willing to take part, participants will be asked to sign the consent form or thumbprint with a witness present if they are not literate. To maximise the safety of participants and study staff, participants with current household cases or symptoms of COVID-19 will be temporarily ineligible to participate and will be requested to revisit the clinic after 21 days. Strict hygiene protocols will be followed and data collection will be paused during periods of high COVID-19 infection. Physical assessments for women will be undertaken by female fieldworkers or with a woman present. Using objective measures to screen for chronic conditions may result in high cognitive and time burden for participants (full clinic protocol approximately 4-hours). To reduce this, participants will be provided meals and refreshments, questionnaires will be administered earlier in the day, and participants will be reimbursed for their time. All participants will be provided with a basic report detailing their BMI, blood pressure, fasting blood glucose, and haemoglobin results. Abnormal physical assessment or biochemical results will be explained to participants by the APCAPS medical officer, who will refer participants to local government or charitable healthcare services for confirmatory diagnoses and treatment.

Dissemination

We will publish our findings in open-access and peer-reviewed journals and disseminate them through scientific conferences and social media accounts of the APCAPS cohort (twitter: @apcaps_study) and team members. We will share summaries of our findings with village leaders, the local community, and the state health department.

Data sharing

We strongly encourage external researchers to use APCAPS data for multimorbidity or other health research. Data will be held under embargo for the study team to conduct the above analyses for one year following the database lockdown. Thoroughly de-identified quantitative and qualitative data will be made available to external researchers following approval of a collaboration request form by the APCAPS executive committee. Meta-data and data collection instruments will be published on the APCAPS website alongside details of existing APCAPS data (www.apcaps.lshtm.ac.uk) and disseminated via social media and networks of the research team. Please contact apcaps.crf@gmail.com with data queries.

References

For peer review only

1. The Academy of Medical Sciences. Multimorbidity: a priority for global health research | The Academy of Medical Sciences [Internet]. 2018. Available from: https://acmedsci.ac.uk/policy/policy-projects/multimorbidity
2. Gupta P, Prabhakaran D, Mohan S. Multimorbidity or multiple long-term conditions: need for bridging the evidence & care gaps to address an emerging priority public health issue in India. Indian J Med Res. 2022;
3. Puri P, Singh SK, Pati S. Temporal dynamics, patterns and correlates of single and multimorbidity in India, 1994–2018. J Multimorbidity Comorbidity. 2021;11:263355652110627.
4. MOSPI. Key Indicators of Social Consumption in India : Health. 2019.
5. Pati S, Agrawal S, Swain S, Lee JT, Vellakkal S, Hussain MA, et al. Non communicable disease multimorbidity and associated health care utilization and expenditures in India: cross-sectional study. BMC Health Serv Res. 2014;14:451.
6. Arokiasamy P, Uttamacharya, Jain K. Multi-morbidity, functional limitations, and self-rated health among older adults in India: Cross-sectional analysis of LASI pilot survey, 2010. SAGE Open. 2015;5(1).
7. Pati S, Swain S, Hussain MA, Van Den Akker M, Metsemakers J, Knottnerus JA, et al. Prevalence and outcomes of multimorbidity in South Asia: A systematic review. BMJ Open. 2015;5(10).
8. Vellakkal S, Millett C, Basu S, Khan Z, Aitsi-Selmi A, Stuckler D, et al. Are estimates of socioeconomic inequalities in chronic disease artefactually narrowed by self-reported measures of prevalence in low-income and middle-income countries? Findings from the WHO-SAGE survey. J Epidemiol Community Health. 2015;69(3):218–25.
9. Gummidi B, Gautam V, John O, Ghosh A, Jha V. Patterns of multimorbidity among a community-based cohort in rural India. J Multimorbidity Comorbidity. 2023;13(8):263355652211496.
10. Garin N, Koyanagi A, Chatterji S, Tyrovolas S, Olaya B, Leonardi M, et al. Global Multimorbidity Patterns: A Cross-Sectional, Population-Based, Multi-Country Study. Journals Gerontol - Ser A Biol Sci Med Sci. 2016;71(2):205–14.
11. Banjare P, Pradhan J. Socio-economic inequalities in the prevalence of multi-morbidity among the rural elderly in Bargarh district of Odisha (India). PLoS One. 2014;9(6).
12. Prados-Torres A, Calderón-Larrañaga A, Hancco-Saavedra J, Poblador-Plou B, Van Den Akker M. Multimorbidity patterns: A systematic review. J Clin Epidemiol. 2014;67(3):254–66.
13. Pati S, Swain S, Metsemakers J, Knottnerus JA, Van Den Akker M. Pattern and severity of multimorbidity among patients attending primary care settings in Odisha, India. PLoS One. 2017;12(9):1–19.
14. Puri P, Singh SK, Pati S. Identifying non-communicable disease multimorbidity patterns and associated factors: a latent class analysis approach. BMJ Open. 2022;12(7):1–12.
15. Marengoni A, Angleman S, Melis R, Mangialasche F, Karp A, Garmen A, et al. Aging with multimorbidity: A systematic review of the literature. Ageing Res Rev [Internet]. 2011;10(4):430–9. Available from: http://dx.doi.org/10.1016/j.arr.2011.03.003
16. Chen H, Goldberg MS, Villeneuve PJ. A systematic review of the relation between long-term exposure to ambient air pollution and chronic diseases. Rev Environ Health. 2008;23(4):243–97.
17. Asogwa OA, Boateng D, Marzà-Florensa A, Peters S, Levitt N, Van Olmen J, et al. Multimorbidity of non-communicable diseases in low-income and middle-income countries: A systematic review and meta-analysis. BMJ Open. 2022;12(1):1–16.
18. Cezard G, McHale CT, Sullivan F, Bowles JKF, Keenan K. Studying trajectories of multimorbidity: A systematic scoping review of longitudinal approaches and evidence. BMJ Open. 2021;11(11):1–19.
19. Rosbach M, Andersen JS. Patient-experienced burden of treatment in patients with multimorbidity – A systematic review of qualitative data. PLoS One. 2017;12(6):1–18.
20. Price ML, Surr CA, Gough B, Ashley L. Experiences and support needs of informal caregivers of people with multimorbidity: a scoping literature review. Psychol Heal [Internet]. 2020;35(1):36–69. Available from: https://doi.org/10.1080/08870446.2019.1626125
21. Smith SM, O'Kelly S, O'Dowd T. GPs' and pharmacists' experiences of managing multimorbidity: A "Pandora's box." Br J Gen Pract. 2010;60(576):285–94.
22. Lall D, Engel N, Devadasan N, Horstman K, Criel B. Challenges in primary care for diabetes and hypertension: An observational study of the Kolar district in rural India. BMC Health Serv Res. 2019;19(1):1–11.
23. Yellapa V, Devadasan N, Krumeich A, Pai NP, Vadnais C, Pai M, et al. How patients navigate the diagnostic ecosystem in a fragmented health system: A qualitative study from India. Glob Health Action [Internet]. 2017;10(1). Available from: https://doi.org/10.1080/16549716.2017.1350452

24. Pati S, Pati S, van den Akker M, Schellevis FG, Sahoo KC, Burgers JS. Managing diabetes mellitus with comorbidities in primary healthcare facilities in urban settings: a qualitative study among physicians in Odisha, India. BMC Fam Pract. 2021;22(1):1–17.
25. Kinra S, Radha Krishna K V, Kuper H, Rameshwar Sarma K V, Prabhakaran P, Gupta V, et al. Cohort profile: Andhra Pradesh Children and Parents Study (APCAPS). Int J Epidemiol. 2014 Oct;43(5):1417–24.
26. Hartung C, Lerer A, Anokwa Y, Tseng C, Brunette W, Borriello G. Open Data Kit: Tools to Build Information Services for Developing Regions. In: Proceedings of the 4th ACM/IEEE International Conference on Information and Communication Technologies and Development. New York, NY, USA: Association for Computing Machinery; 2010. (ICTD '10).
27. Bhan N, Madhira P, Muralidharan A, Kulkarni B, Murthy G, Basu S, et al. Health needs, access to healthcare, and perceptions of ageing in an urbanizing community in India: a qualitative study. BMC Geriatr [Internet]. 2017;17(1):156. Available from: http://bmcgeriatr.biomedcentral.com/articles/10.1186/s12877-017-0544-y
28. Pati S, Hussain MA, Swain S, Salisbury C, Metsemakers JFM, Knottnerus JA, et al. Development and Validation of a Questionnaire to Assess Multimorbidity in Primary Care: An Indian Experience. Biomed Res Int. 2016;2016.
29. Salvi S, Apte K, Madas S, Barne M, Chhowala S, Sethi T, et al. Symptoms and medical conditions in 204 912 patients visiting primary health-care practitioners in India: A 1-day point prevalence study (the POSEIDON study). Lancet Glob Heal [Internet]. 2015;3(12):e776–84. Available from: http://dx.doi.org/10.1016/S2214-109X(15)00152-7
30. Hayter AKM, Jeffery R, Sharma C, Prost A, Kinra S. Community perceptions of health and chronic disease in South Indian rural transitional communities: a qualitative study. Glob Health Action [Internet]. 2015;8:25946. Available from: http://www.pubmedcentral.nih.gov/articlerender.fcgi?artid=4323408&tool=pmcentrez&rendertype=abstract
31. Hurst JR, Agarwal G, Van Boven JFM, Daivadanam M, Gould GS, Wan-Chun Huang E, et al. Critical review of multimorbidity outcome measures suitable for low-income and middle-income country settings: Perspectives from the Global Alliance for Chronic Diseases (GACD) researchers. BMJ Open. 2020;10(9):1–9.
32. Abou-Dakn M, Döhmen C, Wenzel S. Validation of the TONOPORT VI ambulatory blood pressure monitor in adults according to the European Society of Hypertension International Protocol revision 2010. J Hum Hypertens. 2017;31(2):89–92.
33. Takahashi H, Yoshika M, Yokoi T. Validation of three automatic devices for the self-measurement of blood pressure according to the European Society of Hypertension International Protocol revision 2010: the Omron HEM-7130, HEM-7320F, and HEM-7500F. Blood Press Monit. 2015;20(2):92–7.
34. Unger T, Borghi C, Charchar F, Khan NA, Poulter NR, Prabhakaran D, et al. 2020 International Society of Hypertension Global Hypertension Practice Guidelines. Hypertension. 2020;75(6):1334–57.
35. Wang TJ, Evans JC, Benjamin EJ, Levy D, LeRoy EC, Vasan RS. Natural history of asymptomatic left ventricular systolic dysfunction in the community. Circulation. 2003;108(8):977–82.
36. Rose GA. The diagnosis of ischaemic heart pain and intermittent claudication in field surveys. Bull World Health Organ. 1962;27:645–58.
37. Gourie-Devi M, Gururaj G, Satishchandra P, Subbakrishna DK. Neuro-Epidemiological Pilot Survey of an Urban Population in a Developing Country. Neuroepidemiology [Internet]. 1996;15(6):313–20. Available from: https://www.karger.com/DOI/10.1159/000109921
38. Care D, Suppl SS, Brown FM, Bruemmer D, Collins BS, Hilliard ME, et al. 2 . Classification and Diagnosis of Diabetes : Standards of Care in Diabetes — 2023. 2023;46(January):19–40.
39. Bril V, Tomioka S, Buchanan RA, Perkins BA. Reliability and validity of the modified Toronto Clinical Neuropathy Score in diabetic sensorimotor polyneuropathy. Diabet Med. 2009;26(3):240–6.
40. van Doremalen RFM, van Netten JJ, van Baal JG, Vollenbroek-Hutten MMR, van der Heijden F. Validation of low-cost smartphone-based thermal camera for diabetic foot assessment. Diabetes Res Clin Pract [Internet]. 2019;149:132–9. Available from: https://doi.org/10.1016/j.diabres.2019.01.032
41. Herraiz-Adillo Á, Cavero-Redondo I, Álvarez-Bueno C, Pozuelo-Carrascosa DP, Solera-Martínez M. The accuracy of toe brachial index and ankle brachial index in the diagnosis of lower limb peripheral arterial disease: A systematic review and meta-analysis. Atherosclerosis. 2020;315(October):81–92.
42. Bedard C, Liu S, Patterson C, Gerstein H, Griffith L. Systematic review: Can non-mydriatic cameras accurately detect diabetic retinopathy? Diabetes Res Clin Pract [Internet]. 2017;129:154–9. Available from: http://dx.doi.org/10.1016/j.diabres.2017.04.024

43. Arokiasamy P, Parasuraman S, Sekher S V, Lhungdim H. Study on global AGEing and adult health (SAGE) Wave 1India National Report. World Health Organisation; 2013.
44. Hanania NA, Mannino DM, Yawn BP, Mapel DW, Martinez FJ, Donohue JF, et al. Predicting risk of airflow obstruction in primary care: Validation of the lung function questionnaire (LFQ). Respir Med [Internet]. 2010;104(8):1160–70. Available from: http://dx.doi.org/10.1016/j.rmed.2010.02.009
45. Sunyer J, Basagana X, Burney P, Anto JM. International assessment of the internal consistency of respiratory symptoms. Am J Respir Crit Care Med. 2000;162(3 I):930–5.
46. Ranzani OT, Bhogadi S, Milà C, Kulkarni B, Balakrishnan K, Sambandam S, et al. Association of ambient and household air pollution with lung function in young adults in an peri-urban area of South-India: A cross-sectional study. Environ Int. 2022;165(May).
47. Chen LK, Woo J, Assantachai P, Auyeung TW, Chou MY, Iijima K, et al. Asian Working Group for Sarcopenia: 2019 Consensus Update on Sarcopenia Diagnosis and Treatment. J Am Med Dir Assoc [Internet]. 2020;21(3):300-307.e2. Available from: https://doi.org/10.1016/j.jamda.2019.12.012
48. Roberts HC, Denison HJ, Martin HJ, Patel HP, Syddall H, Cooper C, et al. A review of the measurement of grip strength in clinical and epidemiological studies: Towards a standardised approach. Age Ageing. 2011;40(4):423–9.
49. Moussavi S, Chatterji S, Verdes E, Tandon A, Patel V, Ustun B. Depression, chronic diseases, and decrements in health: results from the World Health Surveys. Lancet. 2007;370(9590):851–8.
50. Neufeld LM, Larson LM, Kurpad A, Mburu S, Martorell R, Brown KH. Hemoglobin concentration and anemia diagnosis in venous and capillary blood: biological basis and policy implications. Ann N Y Acad Sci. 2019;1450(1):172–89.
51. De Man J, Absetz P, Sathish T, Desloge A, Haregu T, Oldenburg B, et al. Are the PHQ-9 and GAD-7 Suitable for Use in India? A Psychometric Analysis. Front Psychol. 2021;12(May):1–14.
52. Endsley P, Weobong B, Nadkarni A. Psychometric properties of the AUDIT among men in Goa, India. Asian J Psychiatr [Internet]. 2017;29(2017):54–8. Available from: https://doi.org/10.1016/j.ajp.2017.03.006
53. Prince M, Acosta D, Ferri CP, Guerra M, Huang Y, Jacob KS, et al. A brief dementia screener suitable for use by non-specialists in resource poor settings-the cross-cultural derivation and validation of the brief Community Screening Instrument for Dementia. Int J Geriatr Psychiatry. 2011;26(9):899–907.
54. Ministry of Health and Family Welfare (Government of India). Chapter 3-Case finding & diagnosis strategy [Internet]. Technical and Operational Guidelines for TB Control in India. 2011. Available from: https://tbcindia.gov.in/showfile.php?lid=3216
55. World Health Organization. Tuberculosis prevalence surveys: A handbook [Internet]. 2011. Available from: https://apps.who.int/iris/handle/10665/44481
56. Chadha VK, Anjinappa SM, Dave P, Rade K, Baskaran D, Narang P, et al. Sub-national TB prevalence surveys in India, 2006-2012: Results of uniformly conducted data analysis. PLoS One. 2019;14(2):2006–12.
57. World Health Organization. WHO Covid-19: Case Definitions [Internet]. 2020. Available from: WHO/2019-nCoV/Surveillance_Case_Definition/2020.2
58. World Health Organization. A clinical case definition of post COVID-19 condition by a Delphi consensus [Internet]. 2021. Available from: WHO/2019-nCoV/Post_COVID-19_condition/Clinical_case_definition/2021.1
59. Zulewski H, Müller B, Exer P, Miserez AR, Staub JJ. Estimation of tissue hypothyroidism by a new clinical score: Evaluation of patients with various grades of hypothyroidism and controls. J Clin Endocrinol Metab. 1997;82(3):771–6.
60. World Health Organization. Goitre as a determinant of the prevalence and severity of iodine deficiency disorders in populations. 2014.
61. Bhatia SJ, Reddy DN, Ghoshal UC, Jayanthi V, Abraham P, Choudhuri G, et al. Epidemiology and symptom profile of gastroesophageal reflux in the Indian population: Report of the Indian Society of Gastroenterology Task Force. Indian J Gastroenterol. 2011;30(3):118–27.
62. International Institute for Population Sciences (IIPS). Longitudinal Ageing Study in India (LASI) Wave 1, 2017-18, India Report. Mumbai; 2020.
63. Biobank UK. UK Biobank Pain web questionnaire. 2020;(December):1–29.
64. Aziz Q, Barke A, Bennett MI, Benoliel R, Cohen M, Evers S, et al. A classification of chronic pain for ICD-11. Pain. 2015;156(6):1003–7.
65. Fried LP, Tangen CM, Walston J, Newman AB, Hirsch C, Gottdiener J, et al. Frailty in Older Adults: Evidence for a Phenotype. Journals Gerontol Ser A Biol Sci Med Sci. 2001;56(3):M146–57.

For peer review only

66. Matsuzaki M, Sullivan R, Ekelund U, Krishna KR, Kulkarni B, Collier T, et al. Development and evaluation of the Andhra Pradesh Children and Parent Study Physical Activity Questionnaire (APCAPS-PAQ): A cross-sectional study. BMC Public Health [Internet]. 2016;16(1):1–9. Available from: http://dx.doi.org/10.1186/s12889-016-2706-9
67. Lee EH. Review of the psychometric evidence of the perceived stress scale. Asian Nurs Res (Korean Soc Nurs Sci) [Internet]. 2012;6(4):121–7. Available from: http://dx.doi.org/10.1016/j.anr.2012.08.004
68. Groce NE, Mont D. Counting disability: emerging consensus on the Washington Group questionnaire. Lancet Glob Heal [Internet]. 2017;5(7):e649–50. Available from: http://dx.doi.org/10.1016/S2214-109X(17)30207-3
69. Mactaggart I, Kuper H, Murthy GVS, Oye J, Polack S. Measuring disability in population based surveys: The interrelationship between clinical impairments and reported functional limitations in Cameroon and India. PLoS One. 2016;11(10):1–18.
70. Irace AL, Sharma RK, Reed NS, Golub JS. Smartphone-Based Applications to Detect Hearing Loss: A Review of Current Technology. J Am Geriatr Soc. 2021;69(2):307–16.
71. Renda L, Selçuk ÖT, Eyigör H, Osma Ü, Yılmaz MD. Smartphone based audiometric test for confirming the level of hearing; is it useable in underserved areas? J Int Adv Otol. 2016;12(1):61–6.
72. Aremu S. Evaluation of the hearing test pro application as a screening tool for hearing loss assessment. Niger Med J. 2018;59(5):55.
73. Bright T, Mactaggart I, Kuper H, Murthy G V., Polack S. Prevalence of Hearing Impairment in Mahabubnagar District, Telangana State, India. Ear Hear. 2019;40(1):204–12.
74. World Health Organization. Verbal autopsy standards: ascertaining and attributing causes of death tool. 2022.
75. Coates, J., Swindale, A., Bilinsky P. Household Food Insecurity Access Scale (HFIAS) for Measurement of Household Food Access: Indicator Guide (v.3). Washington, D.C.; 2007.
76. Bowen L, Venkatsubbareddy Bharathi A, Kinra S, DeStavola B, Ness A, Ebrahim SD. Development and evaluation of a semi-quantitative food frequency questionnaire for use in urban and rural India. Asia Pac J Clin Nutr. 2012;21(3):355–60.
77. ECRHS. European Community Respiratory Health Survey: Questionnaires and protocols [Internet]. 2018 [cited 2023 Jan 5]. Available from: https://www.ecrhs.org/questionnaires-and-protocols
78. Kroenke K, Spitzer RL, Williams JBW. The PHQ-9: Validity of a brief depression severity measure. J Gen Intern Med. 2001;16(9):606–13.
79. Spitzer RL, Kroenke K, Williams JBW, Löwe B. A brief measure for assessing generalized anxiety disorder: The GAD-7. Arch Intern Med. 2006;166(10):1092–7.
80. Organization WH. AUDIT: The alcohol use disorders identification test: Guidelines for use in primary health care. World Health Organization; 2001.
81. Cohen S, Williamson G. Perceived stress in a probability sample of the United States [Internet]. Vol. 13, The Social Psychology of Health. 1988. p. 31–67. Available from: http://doi.apa.org/psycinfo/1988-98838-002
82. Washington Group Short Set on Functioning (WG-SS) [Internet]. 2022. Available from: https://www.washingtongroup-disability.com/question-sets/wg-short-set-on-functioning-wg-ss/
83. Jyani G, Sharma A, Prinja S, Kar SS, Trivedi M, Patro BK, et al. Development of an EQ-5D Value Set for India Using an Extended Design (DEVINE) Study: The Indian 5-Level Version EQ-5D Value Set. Value Heal [Internet]. 2022; Available from: https://doi.org/10.1016/j.jval.2021.11.1370
84. Lamb SE, Jørstad-Stein EC, Hauer K, Becker C. Development of a common outcome data set for fall injury prevention trials: The Prevention of Falls Network Europe consensus. J Am Geriatr Soc. 2005;53(9):1618–22.
85. HearingTest app [Internet]. Available from: https://www.e-audiologia.pl/HearingTest/
86. Sorensen TB, Wilson R, Gregson J, Shankar B, Dangour AD, Kinra S. Is night-time light intensity associated with cardiovascular disease risk factors among adults in early-stage urbanisation in South India? A cross-sectional study of the Andhra Pradesh Children and Parents Study. BMJ Open. 2020;10(11):1–11.
87. Sanchez M, Ambros A, Milà C, Salmon M, Balakrishnan K, Sambandam S, et al. Development of land-use regression models for fine particles and black carbon in peri-urban South India. Sci Total Environ [Internet]. 2018;634:77–86. Available from: https://doi.org/10.1016/j.scitotenv.2018.03.308
88. Shi D, Lin Z, Wang W, Tan Z, Shang X, Zhang X, et al. A Deep Learning System for Fully Automated Retinal Vessel Measurement in High Throughput Image Analysis. Front Cardiovasc Med. 2022;9(March):1–12.

For peer review only - http://bmjopen.bmj.com/site/about/guidelines.xhtml

89. May CR, Eton DT, Boehmer K, Gallacher K, Hunt K, MacDonald S, et al. Rethinking the patient: using Burden of Treatment Theory to understand the changing dynamics of illness. BMC Health Serv Res. 2014;14:281.

90. Braun V, Clarke V. Can I use TA? Should I use TA? Should I not use TA? Comparing reflexive thematic analysis and other pattern-based qualitative analytic approaches. Couns Psychother Res. 2021;21(1):37–47.

Author statement

JL, SKu, PM, HM, OK, SS, BK and SK were involved in conception of the study. JL, SKu, PM, HM, SB, SA, NB, WS, AS, OK, GI, SKK, RK, SS, TD, BK and SK were involved in design and development of the study protocol. All authors substantially contributed and commented on the manuscript and approved the final version.

Conflicts of interest

None.

Funding

This work was primarily supported by the UK Medical Research Council (MRC) grant number MC_PC_MR/T038292/1. This research was also part-funded by MRC grant number MR/V001221/1, the Nagasaki University "Doctoral Program for World-leading Innovative and Smart Education" for Global Health, KENKYU SHIDO KEIHI, and in-kind support from ICMR-NIN.

Acknowledgments

We would like to acknowledge and thank the various researchers and practitioners in each partner institution and Ranga Reddy healthcare system that advised on elements of the study protocol, village leaders and local stakeholders for supporting and facilitating data collection, ICMR-NIN for permitting use of their facilities for data collection, the hard-working field team, and most imperatively, the participants who kindly gave us their time.

Figure legend

Figure 1: Overview of feasible analyses examining the determinants, burden and consequences of multimorbidity using previously collected (2010-12) and planned (2022-23) APCAPS data.

For peer review only

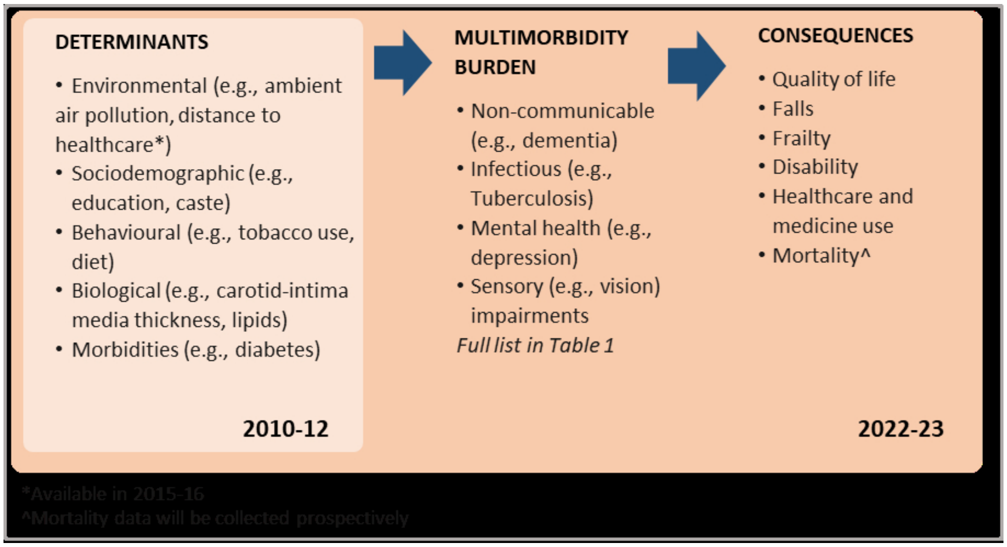

Figure 1: Overview of feasible analyses examining the determinants, burden and consequences of multimorbidity using previously collected (2010-12) and planned (2022-23) APCAPS data.

392x211mm (59 x 59 DPI)

BMJ Open

Burden, determinants, consequences, and care of multimorbidity in rural and urbanising Telangana, India: Protocol for a mixed-methods study within the APCAPS Cohort

Journal:	*BMJ Open*
Manuscript ID	bmjopen-2023-073897.R1
Article Type:	Protocol
Date Submitted by the Author:	30-Oct-2023
Complete List of Authors:	Lieber, Judith; London School of Hygiene and Tropical Medicine Faculty of Epidemiology and Public Health,<br>Banjara, Santosh Kumar; National Institute of Nutrition<br>Mallinson, Poppy; London School of Hygiene and Tropical Medicine Faculty of Epidemiology and Population Health, Department of Non-communicable Disease Epidemiology<br>Mahajan, Hemant; National Institute of Nutrition<br>Bhogadi, Santhi; Public Health Foundation of India<br>Addanki, Srivalli; Public Health Foundation of India<br>Birk, Nick; London School of Hygiene and Tropical Medicine Faculty of Epidemiology and Public Health, Department of Non-communicable Disease Epidemiology<br>Song, Wenbo; London School of Hygiene and Tropical Medicine Faculty of Epidemiology and Public Health; Nagasaki University<br>Shah, Anoop ; London School of Hygiene & Tropical Medicine, Centre for Global Chronic Conditions, Faculty of Epidemiology and Population Health, Department of Non-communicable Disease Epidemiology,<br>Kurmi, Om ; Coventry University<br>Iyer, Gowri; Indian Institute of Public Health Hyderabad<br>Sureshkumar, K; Public Health Foundation of India, SACDIR; London School of Hygiene & Tropical Medicine, International Center for Evidence in Disability<br>Kishore Galla, Raghu; Enact Health Private Limited<br>Sadanand, Shilpa; Indian Institute of Public Health Hyderabad<br>Dasi, Teena; National Institute of Nutrition<br>Kulkarni, Bharati; Indian Council of Medical Research; National Institute of Nutrition<br>Kinra, Sanjay; London School of Hygiene and Tropical Medicine Faculty of Epidemiology and Public Health
**Primary Subject Heading**:	Epidemiology
Secondary Subject Heading:	Epidemiology, Diabetes and endocrinology, Cardiovascular medicine, Global health, Mental health
Keywords:	EPIDEMIOLOGIC STUDIES, Aging, Cardiac Epidemiology < CARDIOLOGY, Dementia, General diabetes < DIABETES & ENDOCRINOLOGY, Risk

For peer review only - http://bmjopen.bmj.com/site/about/guidelines.xhtml

	Factors

SCHOLARONE™
Manuscripts

Burden, determinants, consequences, and care of multimorbidity in rural and urbanising Telangana, India: Protocol for a mixed-methods study within the APCAPS Cohort

Judith Lieber[1]*, Santosh Kumar Banjara[2]*, Poppy AC Mallinson[1], Hemant Mahajan[2], Santhi Bhogadi[3], Srivalli Addanki[3], Nick Birk[1], Wenbo Song[1], Anoop Shah[1], Om Kurmi[4], Gowri Iyer[5], Sureshkumar Kamalakannan[1,5], Raghu Kishore Galla[6], Shilpa Sadanand[5], Teena Dasi[2], Bharati Kulkarni[2,7], Sanjay Kinra[1]

* Contributed equally.

1 – London School of Hygiene and Tropical Medicine, London, WC1E 7HT, UK

2 – National Institute of Nutrition, Indian Council of Medical Research, Hyderabad 50007, India

3 – Indian Institute of Public Health, Bengaluru, 560070, India

4 – Coventry University, Coventry, CV1 5FB, UK

5 - Indian Institute of Public Health, Hyderabad 500033, India

6 – Enact Health Private Limited, Hyderabad, 500018, India

7 – Indian Council of Medical Research, New Delhi, 110029, India

Contact details:

Judith.lieber1@lshtm.ac.uk; drsantoshkumar999@gmail.com; poppy.mallinson@lshtm.ac.uk; hemant.mahajan.84@gmail.com; kammilisanthi.mms@gmail.com; srivalli.addanki@gmail.com; Nick.Birk@lshtm.ac.uk; Wenbo.song@lshtm.ac.uk; Anoop.shah@lshtm.ac.uk; omkurmi@googlemail.com; gowri.iyer@iiphm.org; Suresh.Kumar@lshtm.ac.uk; raghukishoregalla@gmail.com; shilpa848@gmail.com; teena.dasi@icmr.gov.in; dr.bharatikulkarni@gmail.com; sanjay.kinra@lshtm.ac.uk

Corresponding author: Dr Judith Lieber; Judith.lieber1@lshtm.ac.uk; +44 (0)20 7927 2482; London School of Hygiene and Tropical Medicine, London, WC1E 7HT, UK.

Keywords

Risk factors, epidemiologic studies, aging, cardiac epidemiology, dementia, diabetes.

Abstract

Introduction: The epidemiological and demographic transitions are leading to a rising burden of multimorbidity (co-occurrence of two or more chronic conditions) worldwide. Evidence on the burden, determinants, consequences, and care of multimorbidity in rural and urbanising India is limited, partly due to a lack of longitudinal and objectively measured data on chronic health conditions. We will conduct a mixed-methods study nested in the prospective Andhra Pradesh Children and Parents' Study (APCAPS) cohort to develop a data resource for understanding the epidemiology of multimorbidity in rural and urbanising India and developing interventions to improve the prevention and care of multimorbidity.

Methods and analysis: We aim to recruit 2,100 APCAPS cohort members aged 45-plus who have clinical and lifestyle data collected during a previous cohort follow-up (2010-12). We will screen for locally prevalent non-communicable, infectious, and mental health conditions, alongside cognitive impairments, disabilities, and frailty, using a combination of self-reported clinical diagnosis,

For peer review only

symptom-based questionnaires, physical examinations, and biochemical assays. We will conduct in-depth interviews with people with varying multimorbidity clusters, their informal carers, and local healthcare providers. De-identified data will be made available to external researchers.

Ethics and dissemination: The study has received approval from the ethics committees of the National Institute of Nutrition and Indian Institute of Public Health Hyderabad, India, and the London School of Hygiene and Tropical Medicine, UK. Meta-data and data collection instruments will be published on the APCAPS website alongside details of existing APCAPS data and the data access process (www.apcaps.lshtm.ac.uk).

Strengths and limitations of the study

- Open-access multimorbidity resource of 2,100 adults aged 45-plus participating in the community-based APCAPS cohort in rural and urbanising Telangana, India, with scope to conduct longitudinal analyses to examine biological, behavioural, sociodemographic, and environmental risk factors for multimorbidity, multimorbidity progression and consequences.
- A broad range of locally prevalent chronic conditions screened using objective measures to estimate the burden of multimorbidity, overcoming the limitations of self-reporting.
- A qualitative approach to explore the lived experience of people with multimorbidity and their informal carers and identify current gaps in care to guide intervention and policy development.
- Statistical power may be insufficient to examine some less prevalent clusters of multimorbidity.
- The study population is based in a defined geographical area that does not represent the entire population of India and may not capture multimorbidity clusters specific to other Indian settings.

Word count: 3999 (not including tables, references, abstract, headings, figures).

For peer review only

Introduction

Multimorbidity, defined as the co-occurrence of two or more conditions of long-duration ('chronic') in an individual, is rising globally(1). Multimorbidity is associated with poor quality of life, disability, mortality, and healthcare use, which significantly strain health systems(1). In rural India, where 65% of the Indian population reside, socioeconomic developments and globalisation are introducing new risk factors for chronic conditions (e.g., air pollution) alongside prevailing poverty-related risks (e.g., undernutrition). The rising prevalence of non-communicable diseases alongside an ongoing burden of infectious disease is leading to a significant rise in multimorbidity(2,3). Although a system for primary care exists, the majority of outpatient health services are provided by the private sector(4). Lack of a robust system of primary care leaves people with multimorbidity highly vulnerable to catastrophic health expenditure and poor health and social outcomes(5,6). Evidence on the burden, determinants, consequences, and gaps in care of multimorbidity is required to improve the prevention and care of multimorbidity in rural and urbanising India and other settings undergoing similar trends(2).

Current evidence on the burden of multimorbidity in rural and urbanising India is limited. Incidence data are lacking, while prevalence estimates have fluctuated widely (ranging from 9 to 59%) owing to a frequent reliance on self-reported disease, which is known to grossly underestimate true disease prevalence in these settings(6–9). There is some evidence that multimorbidity clusters in India mirror those reported in high-income countries, specifically, clustering of cardio-respiratory (angina, asthma, chronic obstructive pulmonary disease (COPD)), metabolic (diabetes, obesity, hypertension) and mental-articular (arthritis and depression) conditions(10–14). However, multimorbidity clusters specific to rural and urbanising India may have not been identified as several locally prevalent conditions (e.g., anaemia) were not measured in prior studies(9–11,13,14).

Longitudinal studies from high-income countries have identified several risk factors (e.g., air pollution) associated with multiple chronic conditions (e.g., cardio-respiratory, cancers) and social determinants of multimorbidity(15,16). However, longitudinal evidence on the risk-factors for multimorbidity is critically lacking in India and other low- and middle-income countries (LMICs)(17,18). While a few longitudinal studies from India have investigated the risk factors of individual chronic conditions, the effect of these or other common risk factors in rural India (e.g., indoor use of biomass fuel) on multimorbidity has not been examined(7).

Studies in high-income countries have reported the challenges of caring for multimorbidity for the individual as well as their informal carers (e.g., impacted social relationships), and healthcare providers (e.g., lack of clinical guidance for treatment of multiple conditions)(19–21). Studies of individual conditions in India have highlighted long, complex and burdensome care pathways(22,23). However, evidence of the lived experience of multimorbidity for patients and their informal carers is lacking in India, particularly in rural areas. Further, few studies have explored the barriers to care of multimorbidity in India, focusing specifically on diabetes comorbidities(22,24).

Aim and objectives

We aim to develop a longitudinal data resource for examining the epidemiology of multimorbidity in rural and urbanising India and developing interventions to improve the prevention and care of multimorbidity. We envisage that this will also have relevance for other rural and urbanising settings at similar stages in their epidemiological transition. Our objectives are:

1. To estimate the prevalence and incidence of multimorbidity (defined as two or more chronic conditions) and common multimorbidity clusters in rural and urbanising Telangana, India.

For peer review

2. To examine the biological, behavioural, sociodemographic, and environmental risk factors for, and consequences of, multimorbidity.
3. To explore the lived experience of individuals with multimorbidity and their informal carers.
4. To assess how care for multimorbidity is organised in rural and urbanising India and identify the barriers and enablers to high-quality care.

Methods and analysis

Study design

A mixed-methods study in the community-based Andhra Pradesh Children and Parents Study (APCAPS) cohort(25). The qualitative methods (objectives 3-4) will be embedded within the quantitative study (objectives 1-2), using the quantitative data as a sampling frame.

Study population and setting.

APCAPS is a prospective inter-generational cohort that has been incrementally built through long-term follow-up of a nutrition trial (1987-90) conducted in 29 villages (Ranga Reddy district) in the south Indian state of Telangana. The 29 villages of the APCAPS cohort are located 50-100 km from Hyderabad, the state capital, and have urbanised at varying rates, now presenting a mix of rural and urban risk factors. For children born during the original nutrition trial, chronic disease risk factor and outcome data has been collected repeatedly (2003-05 to 2010-12). In 2010-12, similar data was collected for their parents and siblings (N=6,972 total cohort). Figure 1 provides an overview of the longitudinal multimorbidity analyses feasible with the 2010-12 and 2022-23 data.

Inclusion criteria

Adults aged 45 years and above who participated in a prior APCAPS study residing in the study villages or surrounding area are eligible to participate. This group primarily comprises the parents' generation of the cohort plus older siblings of the trial children. While the target population are aged 45-plus, cohort members aged 18-44 are eligible to undergo a limited protocol if they wish to participate.

Exclusion criteria

None.

Quantitative data collection

Figure 1 here.

Quantitative sample

A cohort tracking exercise (April-August 2021) identified N=2,571 APCAPS members eligible to participate (58% women). We anticipate a final sample size of N=2,057 (expected mean age 63 and 56 years for men and women, respectively) based on an estimated 80% follow-up. The proposed sample size will allow us to estimate the 10-year incidence of any multimorbidity (defined as two or more chronic conditions), and incidence of specific common multimorbidity clusters, with a precision of 4% and 3% respectively (assuming a conservative N=700 new cases of multimorbidity and three equal-sized clusters)(10). For analysis of risk factors, we will have statistical power to detect a rate ratio of ~1.25 (assuming an incidence rate of 35/1000 person-years in the unexposed with a 30% risk factor prevalence and 80% power at a 5% significance level).

Quantitative data collection procedures

For peer review only

Following piloting, data collection commenced in February 2022 and will be completed towards the end of 2023. Data collection is occurring village-by-village, with clinics occurring in the study villages or at the central study office (ICMR-National Institute of Nutrition (NIN), Hyderabad), or the household when necessary, e.g., for people with mobility impairments. Questionnaires and physical examinations occur at the clinic, and participants are invited to return the following weekend to provide blood and urine samples. Questionnaire responses and results of most physical assessments are collected by tablet using the Open Data Kit (ODK) platform(26). Other data are extracted directly from the relevant instruments.

Quantitative protocol

The full protocol consists of questionnaires, a physical examination, and collection of urine and fasting blood samples. The screened conditions were selected based on their reported prevalence in India and their importance to the local community(27–30). We are screening for chronic diseases with a combination of self-reported diagnosis by a healthcare professional, symptom-based questionnaires, physical examinations, and biochemical assays, in order to overcome the socioeconomic bias observed when classifying conditions by prior clinical diagnosis in rural India(8) (table 1). The questionnaires cover sociodemographics, behavioural risk factors, self-reported clinical diagnosis of chronic conditions, treatment details, symptom-based screening tools, and impact measures (e.g., quality of life). We limit questions about prior clinical diagnosis to conditions with a higher likelihood of diagnosis in the study population, based on assessment of existing and pilot APCAPS data and discussions with local healthcare providers. The protocol was designed to maximise the scope for longitudinal analyses and to measure key chronic conditions and impact outcomes(31).

The physical assessment includes a detailed examination of cardiovascular physiology, anthropometry and physical functioning assessments, and assessment of diabetes complications (neuropathy and ulceration) for participants reporting a prior diagnosis of diabetes or screening positive for pre-diabetes/diabetes. Individuals with limb or spine deformities are excluded from the anthropometry protocol, and individuals with mobility impairments are excluded from the 6-meter timed walk if they/the fieldworker deem it unsafe. Pregnant women and individuals with a prior diagnosis of diabetes are not requested to fast overnight for blood sampling but are eligible to provide a sample. As this is an ongoing cohort, we plan to prospectively collect mortality data, including cause of death.

Table 1: Screened health conditions and states of poor health, by type of screening measure

Health condition/state	Screening measure		
	Self-reported clinical diagnosis	Symptom-based questionnaire	Physical examination/ biochemical assay
Hypertension	✓		✓
Left ventricular dysfunction/Heart failure*	✓		✓
Coronary heart disease*	✓	✓	
Atrial fibrillation*	✓		✓
Atherosclerosis			✓
Stroke	✓	✓	
Underweight/obesity			✓

For peer review only

Diabetes	✓		✓
Diabetic ulceration	✓		✓
Diabetic neuropathy	✓	✓	✓
Peripheral arterial disease	✓	✓	✓
Retinopathy	✓		✓
Cataracts	✓	✓	
Chronic obstructive pulmonary disease		✓	
Asthma	✓	✓	
Sarcopenia			✓
Arthritis	✓	✓	
Anaemia			✓
Depression		✓	
Anxiety		✓	
Alcohol Use Disorder		✓	
Dementia		✓	
Tuberculosis	✓	✓	
COVID-19	✓	✓	
Thyroid dysfunction	✓	✓	✓
Chronic liver disease	✓		✓
Chronic kidney disease	✓		✓
Peptic ulcer	✓		
Gastro-oesophageal reflux disease	✓	✓	
Cancer	✓		
Poor oral health		✓	
Chronic pain		✓	
Chronic stress		✓	
Frailty		✓	✓
Disability		✓	✓
Vision impairment	✓		✓
Hearing impairment	✓		✓
*Question on prior clinical diagnosis refers to 'heart disease' but does not categorise by condition.			

For peer review only

Validity of quantitative protocol

Hypertension is assessed by seated brachial blood pressure (three readings) with validated oscillometric devices(32–34) (table 2). Left ventricular dysfunction is assessed by 2D-Echocardiography performed by an experienced echo technician following a standardised protocol(35), coronary heart disease symptoms with the validated WHO Rose questionnaire(36), atrial fibrillation with 12-lead ECG, and atherosclerosis with carotid-intima media thickness (CIMT). A stroke is measured with a symptom-based questionnaire developed and commonly used to estimate the community-based prevalence in India(37). Underweight and obesity are assessed with height, weight, and hip and waist circumferences to estimate body mass index (BMI) and waist-to-hip ratio, and bioelectrical impedance to estimate body fat percentage, with all measures taken twice and using the same standardised protocols as earlier APCAPS surveys(25).

Diabetes is screened by glycated haemoglobin (HbA1c) and Fasting Plasma Glucose, both gold-standard diagnostic tests(38). Participants with self-reported diabetes and/or who screen

positive for pre-diabetes or diabetes (based on Fasting Plasma Glucose) are undergoing a diabetic neuropathy examination and assessment for diabetic ulceration. We do not predict advanced neuropathy in the study population due to their relatively young age (average 59 years). As such, we are using the validated modified Toronto Clinical Neuropathy Score (mTCNS) which aims to detect early dysfunction(39). Diabetic foot ulcerations are assessed with infrared thermal imaging, a validated measure(40). In all participants, peripheral arterial disease (PAD) is assessed by toe-brachial index (TBI) which has better sensitivity in people with diabetes (in whom we predict most cases of PAD) versus the more commonly used ankle-brachial index(41). Retinal images are being taken to screen for retinopathy, with two fields (centred on the optic disc and the macula) to improve validity(42). We are using a non-mydriatic camera without pupil dilation to maximise participant comfort. Cataracts are assessed with a symptom-based tool previously used in a nationwide survey in India(43).

To reduce risk of COVID-19 transmission, chronic respiratory conditions (COPD and asthma) are assessed with validated symptom-based questionnaires(44,45) instead of spirometry (used in prior APCAPS surveys(46)). Sarcopenia is defined by muscle strength, physical performance, and appendicular skeletal muscle mass(47). Muscle strength is measured by grip strength using the same instrument and protocol as the 2010-12 APCAPS survey as grip strength estimates are highly sensitive to instrument and protocol used(25,48). In line with the Asian Working Group for Sarcopenia's recommendations, physical functioning is being measured with a 6-meter timed walk at normal speed with a dynamic start, and appendicular skeletal muscle mass with bio-impedance analysis(47). Arthritis is screened with a cross-culturally valid symptom-based questionnaire(49). Anaemia is assessed by haemoglobin concentration of fasting venous blood with an auto-analyser on the day of sample collection (a gold-standard diagnostic test(50)). Mental health conditions (depression, anxiety, and alcohol-use disorder) and dementia are being screened with widely used questionnaires validated for Indian populations(51–53). Due to the relatively low expected prevalence of tuberculosis in the study population, and high resource requirements and participant burden for diagnostic testing, we screen for tuberculosis with a bespoke symptom-based questionnaire. The questions are based on the Government of India's definition of presumptive tuberculosis and WHO guidance for tuberculosis surveys to allow within- and across-country prevalence comparisons respectively(54–56). COVID-19 history and post COVID-19 condition (Long COVID) are assessed with a combination of self-report and symptom-based questions devised for the study based on WHO case definitions(57,58). As we could not identify a validated tool that was sufficiently brief, thyroid dysfunction is assessed with questions developed for the study based on Zulewski's list of symptoms of hypothyroidism(59) and fieldworker's visual assessment of goitre (bespoke protocol based on the WHO case definition of goitre(60)). Liver disease is assessed with a 2D ultrasound to detect liver fibrosis and fatty liver combined with fasting blood biomarkers (table 3). Chronic kidney disease is assessed by estimated Glomerular Filtration Rate (eGFR) based on serum creatinine, weight, age and sex using appropriate equations, and urine albumin to creatinine ratio. Gastro-oesophageal reflux disease (GORD) is assessed with a symptom-based questionnaire developed for Indian populations(61).

As recommended by a recent working group of experts in multimorbidity(1), we are measuring additional states of poor health (poor oral health, chronic pain, frailty, chronic stress) as well as disability. Oral health is assessed with a symptom-based questionnaire of a nationwide survey in India(62). We define chronic pain as persistent or recurring pain lasting more than 3-months (in line with ICD-11), assessed with a questionnaire from the UK Biobank pain survey (as we could not identify a sufficiently brief questionnaire validated in India(63,64)). We conceptualise frailty with the widely used Fried frailty phenotype(65): slowness (measured with the 6-m timed

For peer review only

walk), physical activity (validated APCAPS physical activity questionnaire(66)), weakness (grip strength), weight loss (question devised for the study (tuberculosis symptom questionnaire)), and exhaustion (question in the depression tool, PHQ-9). The short (4-item) version of the cross-culturally validated Perceived Stress Scale is used to assess chronic psychological stress to minimise participant burden(67). As recommended by prior studies in rural India, disability is measured with a validated functional limitation tool previously tested in India(68,69), as well as screening for impairments. Vision impairment (near and distant) is measured with a standard visual acuity test protocol using the Tumbling E LogMAR chart, which is appropriate for people with low literacy. Hearing impairment is assessed with a smartphone-based hearing threshold test using the validated Hearing Test (pro) application (e-audiological.pl)(70). HearingTest has demonstrated good validity at higher frequencies(71,72); presbycusis (characterised by hearing loss at high frequencies) is likely the most common cause of hearing loss in the study population(73). Information on deaths occurring in the cohort will be collected from community health (ASHA) workers, who routinely monitor mortality in the villages, on a 6-monthly prospective basis (project funding permitting). Close contacts of the deceased will be interviewed using the validated WHO verbal autopsy instrument (2022 version) to ascertain cause of death(74).

Table 2: Measures collected in planned study (2022-23), source of questionnaire/instrument and protocol, and availability in prior (2010-2012) APCAPS study.
●-collected with same instrument, ⊖-collected with different instrument, ○-not collected in 2010-12.

Measure	Source of questionnaire/Instrument and protocol	Availability in 2010-12
	**Questionnaires**	
Sociodemographics	APCAPS 2010-12 survey	●
Food insecurity	Household Food Insecurity Access Scale (9-item)(75)	○
Family health history	APCAPS 2010-12 survey	●
Tobacco use	APCAPS 2010-12 survey	●
Alcohol use	Validated APCAPS food frequency questionnaire(76)	●
Diet	Validated APCAPS food frequency questionnaire(76)	●
Physical activity	Validated APCAPS physical activity questionnaire(66)	●
Sleep	APCAPS 2010-12 survey	●
Medical history	APCAPS 2010-12 survey, photos of medicines.	●
CHD symptoms	WHO Rose Questionnaire(36)	●
PAD symptoms	WHO Rose Questionnaire(36)	○
Stroke	Gourie-Devy et al., survey(37)	⊖
Cataracts	SAGE survey	○
Chronic Obstructive Pulmonary Disease	Lung Function Questionnaire(44)	⊖
Asthma	European Community Respiratory Health Survey (short)(77)	⊖
Arthritis	SAGE survey	○
Depression	Patient Health Questionnaire-9 (PHQ-9)(78)	●
Generalised Anxiety Disorder	Generalised Anxiety Disorder-7 (GAD-7)(79)	○
Alcohol Use Disorder	Alcohol Use Disorders Identification Test(80)	○
Dementia	Brief Community Screening Instrument for Dementia(53)	○
Tuberculosis	Developed for study (Government of India’s case definition of presumptive tuberculosis plus night sweats(54,55))	⊖
COVID-19, post COVID-19 condition	Developed for study (WHO case definitions)(57,58)	○

For peer review only

Hypothyroidism	Developed for study (Zulewski case definition(59))	⊖
Gastro-Oesophageal Reflux Disease	Indian Society of Gastroenterology survey(61)	○
Oral health	LASI survey	○
Chronic pain	UK Biobank survey(63)	○
Chronic stress	Perceived Stress Scale (Short Version)(81)	○
Disability	Washington Group Short Set on Functioning(82)	⊖
Health-related quality of life	EuroQol 5-Dimension Health Questionnaire, 5-level(83)	●
Healthcare use	LASI survey	○
Medicine costs	LASI survey	○
Falls	Developed for study (Prevention of Falls Network Europe case definition(84))	○
Cause of death	2022 WHO Verbal Autopsy Instrument (85)	○
**Physical/ cognitive**		
Brachial blood pressure	Three readings taken in the right arm, sitting position (Omron HEM-7121 and Uscom BP+)	●
Heart structure and function	2D Echocardiogram (Philips CX-50 2D-ultrasound, S5-1 PureWave Cardiac Sector probe)	○
Arrhythmia	Resting ECG (12-lead 12-channel Cardiax ECG)	○
Carotid Intima-Media Thickness	Close to bifurcation (Philips CX-502D-ultrasound, linear probe, Philips QLAB software)	⊖
Arterial stiffness	Three readings taken in the right arm, sitting position (Uscom BP+ supra-systolic central blood pressure monitor)	⊖
Standing height	End of expiration to nearest 1mm (Seca Leicester stadiometer)	●
Weight	Single frequency bioelectrical impedance (BIA) scale (TANITA BC418 M57NA) or, if unable to undergo BIA, digital scales to the nearest 0.1kg (Seca 899)	●
Body composition	Single frequency BIA scale (TANITA BC418 M57NA), two measures taken wearing light clothes and bare feet.	●
Waist circumference	Narrowest point of the waist from the front (ADE non-stretch metallic tape)	●
Hip circumference	Maximum extension of buttock from the side (ADE non-stretch metallic tape)	●
Diabetic ulceration	Infrared images of both feet following 15-minute rest (CAT-S61 smartphone with FLIR thermal imaging camera)	○
Diabetes neuropathy	Sensory assessment of the foot and symptom-based questionnaire (mTCNS)(39)	○
Toe brachial index	Ratio of brachial and toe pressures (three measures), photoplethysmography method (Vicorder, Skidmore Medical), taken in a supine position after 5-minute rest.	○
Retinopathy	Retinal images centred on the optic nerve and macula taken in a darkened room without dilation (Bosch digital fundus camera (non-mydriatic))	○
Gait speed and characteristics	6-meter timed walk at normal speed with dynamic start and no deceleration with a waist-worn accelerometer (Actigraph)	○
Grip strength	Standing position, elbows at 20° angle, two measures in both hands (Lafayette Hand-held Dynamometer 78010)	●

For peer review only - http://bmjopen.bmj.com/site/about/guidelines.xhtml

Goitre	Visual assessment (WHO goitre case definition)(60)	○
Liver fibrosis	Ultrasound of right lobe with peri-portal vasculature visible (Philips CX-50 2D-ultrasound, C5-1 PureWave Convex probe)	○
Vision impairment	Tumbling E LogMAR chart (4m and 40cm distance) (Precision Vision)	○
Hearing impairment	Phone-based pure-tone audiometry (Hearing Test (pro) application (e-audiological.pl)(86), Sennheiser HD 458 BT headphones)	○
Retrieval fluency	Maximum animals named in 1-minute (animal naming test)	○
**Environment**		
Village-level urbanisation	Night-time light intensity remote sensing data(87)	●
Air pollution	PM2.5 and black carbon prediction models(88)	●
Built environment	Shops selling food, tobacco & alcohol, health services, educational services, advertisements, public transport and walkability, physical activity (surveyed and geolocated)	⊖*
APCAPS=Andhra Pradesh Children and Parents Study, CHD = Coronary Heart Disease, PAD = Peripheral Arterial Disease, PM2.5 = Particulate Matter 2.5, LASI = Longitudinal Aging Study in India, SAGE = Study on Global Ageing and Adult Health, WHS = World Health Survey, WHO = World Health Organisation. * Surveyed and geolocated in 2016, not updated in 2022-23.		

Sample collection and processing

Participants are asked to fast overnight (at least 8 hours) and rest quietly for 10 minutes before venous blood samples (up to 20ml) are collected from their preferred arm by a trained phlebotomist (table 3). Blood are collected into appropriate vacutainers (2 plain, 1 EDTA, 1 Na-F EDTA, Becton Dickinson, New Jersey, United States) then centrifuged and aliquoted in situ within 1-4 hours. Samples are kept on ice until being transferred to -80°C freezers at the central laboratory, within six hours of collection. Participants are given a urine collection container the day before their scheduled clinic and instructed to collect a mid-stream urine sample from their first urination the next morning. Samples are kept on ice during the clinic and transferred to the central laboratory to be centrifuged (to remove cells/debris), aliquoted, and stored at -80°C that day.

Sample analysis

On the same day as sample collection, the following assays are being conducted at the central laboratory (ICMR-NIN, Hyderabad, India): Complete blood count using autoanalyzer (Horiba ABX Micros 60 OT, Japan) and plasma glucose using GOD-POD method (Randox laboratories India Pvt Ltd) (table 3). Appropriate 2-level or 3-level quality controls are in place as provided by the manufacturers. Other assays will be conducted in batches using frozen serum or plasma or urine samples.

Table 3: Biological samples collected in planned study (2022-23), protocol/assay, and availability in prior (2010-2012) APCAPS study.
●-collected with same instrument, ⊖-collected with different instrument, ○-not collected in 2010-12.

Stored biological samples and assays		
**Measure**	**Protocol/assay**	**Availability in 2010-12**
Blood	Venous blood (overnight fast), processed on the same day (plasma and serum aliquots) and stored at -80C)	●

For peer review only

Urine	First urination of the day, mid-stream, processed on the same day and stored at -80C	○
Haemoglobin	Auto-analyser (ABX Micros 60 – HORIBA)	⊖
Fasting Blood Glucose	GOD-POD method (Randox laboratories, India Pvt Ltd)	●
HbA1c	To be confirmed	○
Serum lipids (HDL, TC, Trig)	To be confirmed	●
Liver function tests (ALT, AST, ALP Bilirubin)	To be confirmed	●
Serum creatinine	To be confirmed	●
Urine albumin to creatinine ratio	To be confirmed	○
HbA1c = glycated haemoglobin, HDL = high-density lipoprotein, TC = total cholesterol, Trig = triglycerides, ALT = alanine transaminase, AST = aspartate aminotransferase, ALP = alkaline phosphatase.		

Quality of quantitative data

Fieldworkers were trained in consent procedures, ODK, survey interviewing, and the anthropometry examination (following the same protocols as prior APCAPS surveys) by the experienced APCAPS supervisory team (10-15 years working with the cohort). For measures not collected in prior studies (table 2), fieldworkers were either trained by coinvestigators (in their respective fields) or external experts. Developers of the mTCNS instrument and a neurophysician trained fieldworkers to conduct the neuropathy assessment(39). A clinical ophthalmologist trained fieldworkers to collect retinal images, provided quality control feedback on initial images collected and refresher training. Experienced technicians trained in 2D-Echo are undertaking the echocardiogram, liver scan, and CIMT. A team of experienced phlebotomists and biotechnicians are collecting, aliquoting and storing biological samples. Prior to data collection, the full protocol (questionnaires and physical examinations) was piloted in the community.

To maximise reliability, rigorous protocols have been developed, each measure is collected by the same 2-3 fieldworkers to minimise inter-rater variability, and instruments are calibrated at daily, weekly, monthly and/or yearly intervals based on relevant guidelines. The supervisory team observe, evaluate, and feedback on protocol implementation in the field using a quality control proforma. To minimise data errors, data collected through the ODK platform restricts implausible values, and other data is extracted directly from instruments when feasible. Data quality reports that describe missing data, variable distributions, and questionnaire completion times are assessed by the research team fortnightly. On an ongoing basis, quality of echocardiograms, ECG traces, and CIMT images are examined by an experienced cardiologist and the retinal images with an automated tool(89). The protocol is repeated in a random 5% of participants 2-4 weeks following initial data collection, intra-class correlation coefficients (ICCs) are estimated for key continuous variables (e.g., anthropometrics) as the study is ongoing, and refresher training conducted if ICCs indicate decreasing reliability.

To ensure correct data linkage, participants' personal details are used to link them to their unique APCAPS identifiers (ID) upon attending the clinic. Each participant has a folder with a QR code linked to their APCAPS ID to track protocol completion, minimise missing data, link each

For peer review only

dataset to the correct participant, and link incoming data to existing APCAPS data. Sample vials are labelled with QR codes linked to APCAPS IDs. During data cleaning, age and sex will be cross-checked across datasets, and discrepancies will be compared against raw data to ensure correct linkage.

Data analysis

While the study team have prespecified analyses, external researchers can use APCAPS data to assess their own research questions(25) (see data sharing). Assessment of the burden of multimorbidity will include descriptive statistics of the prevalence and (where possible) 10-year cumulative incidence of multimorbidity. Multimorbidity outcomes will be defined as both count and clusters of conditions (our primary outcome), conjointly identified with two commonly used clustering methods (latent class analysis (LCA) and multiple correspondence analysis (MCA))(90). Risk factors (e.g., environmental, sociodemographic, behavioural, and biological) and consequences (e.g., health-related quality of life) of multimorbidity will be assessed by multiple logistic or ordinal logistic regression modelling as appropriate (figure 1), adjusted for appropriate covariates and using multiple imputation to complete missing data values where necessary.

Limitations

Comparable baseline data for incidence analyses will be lacking for some conditions. However, for several age-related conditions (e.g., dementia, sarcopenia) this is unlikely to bias the incidence estimates notably due to their low prevalence in under-50s (mean cohort age 49 years in 2010-12). Other conditions that may have manifested at baseline (e.g., generalised anxiety disorder) can be excluded from incidence analyses. Self-reported clinical diagnoses will not be validated against medical notes due to inconsistent record keeping by participants. To reduce participant burden, screening instruments were selected to balance validity in the study population and time taken to complete. As a result, some conditions were not assessed with gold-standard measures (e.g., tuberculosis).

Qualitative data collection

Following quantitative data collection, we will use qualitative methods to explore the lived experience of individuals with multimorbidity and their informal carers, to understand how healthcare is organised for people with multimorbidity, and to identify the barriers and enablers to high-quality care of people with multimorbidity. Participants will include individuals with multiple diagnosed conditions, their informal carers (defined as someone who helps them with their health regularly), and local healthcare providers involved in the diagnosis and management of chronic conditions. This is guided by the Burden of Treatment Theory, which models how patients, their social networks, and the healthcare system interact to address the burden of treatment, a key issue in multimorbidity(19,91).

Qualitative sample

We will use a purposive sampling strategy aiming for maximum variation in clusters of diagnosed conditions. The quantitative data will be analysed using clustering approaches to identify the most common concordant, discordant, and complex multimorbidity clusters (approximately N=15)(92). Around half of the interviewees will be asked to refer an informal carer to participate. We will also aim for maximum variation in healthcare provider type, including doctors in local primary health centres and district hospitals, registered medical practitioners, and pharmacists (approximately N=15). Transcripts will be reviewed as data collection is ongoing, further interviews may be undertaken to explore issues being raised by participants.

For peer review only

Qualitative methods and analysis

We will undertake in-depth, one-to-one interviews with participants in the local language (Telugu), which will be audio-recorded. The Burden of Treatment Theory, plus existing evidence on diagnosis, management, and impact of chronic conditions in India, will guide development of the topic guides(91). We will use a reflective thematic analysis approach which is appropriate for capturing diverse experiences and developing practice-relevant recommendations(93). We will undertake inductive-deductive coding, guided but not restricted to a coding framework with high-level categories based on the Burden of Treatment Theory(91).

Qualitative data quality

Prior to data collection, topic guides will be piloted in the community to evaluate relevance, comprehension, and length. Interviews will be conducted by experienced qualitative interviewers. To be sensitive to local gender norms and maximise participant comfort, interviewers of the same gender as participants will undertake the interviews in private locations (as feasible) of the participants' choosing. A subset of the early transcripts will be double-coded to clarify code definitions and initiate discussion and interpretations.

Patient and public involvement

The study design was based on insights from in-depth interviews and focus groups with community members and local health providers, who perceived a high burden of a range of cardiometabolic, mental health, and skeletal conditions, and sensory and mobility impairments in the population(27). The study design was discussed with and approved by village leaders.

Ethics

The study protocol and tools have been approved by the ethics committees of ICMR-NIN (CR/1/V/2023) and Indian Institute of Public Health Hyderabad (IIPHH/TRCIEC/189/2018), India, and the London School of Hygiene and Tropical Medicine (21771 /RR/19113), UK. Trained fieldworkers will verbally explain the study information sheet to potential participants; if willing to take part, participants will be asked to sign the consent form or thumbprint with a witness present if they are not literate. To maximise the safety of participants and study staff, participants with current household cases or symptoms of COVID-19 will be temporarily ineligible to participate and will be requested to revisit the clinic after 21 days. Strict hygiene protocols will be followed and data collection will be paused during periods of high COVID-19 infection. Physical assessments for women will be undertaken by female fieldworkers or with a woman present. Using objective measures to screen for chronic conditions may result in high cognitive and time burden for participants (full clinic protocol approximately 4-hours). To reduce this, participants will be provided meals and refreshments, questionnaires will be administered earlier in the day, and participants will be reimbursed for their time. All participants will be provided with a basic report detailing their BMI, blood pressure, fasting blood glucose, and haemoglobin results. Abnormal physical assessment or biochemical results will be explained to participants by the APCAPS medical officer, who will refer participants to local government or charitable healthcare services for confirmatory diagnoses and treatment.

Dissemination

We will publish our findings in open-access and peer-reviewed journals and disseminate them through scientific conferences and social media accounts of the APCAPS cohort (twitter:

For peer review only - http://bmjopen.bmj.com/site/about/guidelines.xhtml

@apcaps_study) and team members. We will share summaries of our findings with village leaders, the local community, and the state health department.

Data sharing

We strongly encourage external researchers to use APCAPS data for multimorbidity or other health research. Data will be held under embargo for the study team to conduct the above analyses for one year following the database lockdown. Thoroughly de-identified quantitative and qualitative data will be made available to external researchers following approval of a collaboration request form by the APCAPS executive committee. Meta-data and data collection instruments will be published on the APCAPS website alongside details of existing APCAPS data (www.apcaps.lshtm.ac.uk) and disseminated via social media and networks of the research team. Please contact apcaps.crf@gmail.com with data queries.

References

1. The Academy of Medical Sciences. Multimorbidity: a priority for global health research | The Academy of Medical Sciences [Internet]. 2018. Available from: https://acmedsci.ac.uk/policy/policy-projects/multimorbidity
2. Gupta P, Prabhakaran D, Mohan S. Multimorbidity or multiple long-term conditions: need for bridging the evidence & care gaps to address an emerging priority public health issue in India. Indian J Med Res. 2022;
3. Puri P, Singh SK, Pati S. Temporal dynamics, patterns and correlates of single and multimorbidity in India, 1994–2018. J Multimorbidity Comorbidity. 2021;11:263355652110627.
4. MOSPI. Key Indicators of Social Consumption in India : Health. 2019.
5. Pati S, Agrawal S, Swain S, Lee JT, Vellakkal S, Hussain MA, et al. Non communicable disease multimorbidity and associated health care utilization and expenditures in India: cross-sectional study. BMC Health Serv Res. 2014;14:451.
6. Arokiasamy P, Uttamacharya, Jain K. Multi-morbidity, functional limitations, and self-rated health among older adults in India: Cross-sectional analysis of LASI pilot survey, 2010. SAGE Open. 2015;5(1).
7. Pati S, Swain S, Hussain MA, Van Den Akker M, Metsemakers J, Knottnerus JA, et al. Prevalence and outcomes of multimorbidity in South Asia: A systematic review. BMJ Open. 2015;5(10).
8. Vellakkal S, Millett C, Basu S, Khan Z, Aitsi-Selmi A, Stuckler D, et al. Are estimates of socioeconomic inequalities in chronic disease artefactually narrowed by self-reported measures of prevalence in low-income and middle-income countries? Findings from the WHO-SAGE survey. J Epidemiol Community Health. 2015;69(3):218–25.
9. Gummidi B, Gautam V, John O, Ghosh A, Jha V. Patterns of multimorbidity among a community-based cohort in rural India. J Multimorbidity Comorbidity. 2023;13(8):263355652211496.
10. Garin N, Koyanagi A, Chatterji S, Tyrovolas S, Olaya B, Leonardi M, et al. Global Multimorbidity Patterns: A Cross-Sectional, Population-Based, Multi-Country Study. Journals Gerontol - Ser A Biol Sci Med Sci. 2016;71(2):205–14.
11. Banjare P, Pradhan J. Socio-economic inequalities in the prevalence of multi-morbidity among the rural elderly in Bargarh district of Odisha (India). PLoS One. 2014;9(6).
12. Prados-Torres A, Calderón-Larrañaga A, Hancco-Saavedra J, Poblador-Plou B, Van Den Akker M. Multimorbidity patterns: A systematic review. J Clin Epidemiol. 2014;67(3):254–66.
13. Pati S, Swain S, Metsemakers J, Knottnerus JA, Van Den Akker M. Pattern and severity of multimorbidity among patients attending primary care settings in Odisha, India. PLoS One. 2017;12(9):1–19.
14. Puri P, Singh SK, Pati S. Identifying non-communicable disease multimorbidity patterns and associated factors: a latent class analysis approach. BMJ Open. 2022;12(7):1–12.
15. Marengoni A, Angleman S, Melis R, Mangialasche F, Karp A, Garmen A, et al. Aging with multimorbidity: A systematic review of the literature. Ageing Res Rev [Internet]. 2011;10(4):430–9. Available from: http://dx.doi.org/10.1016/j.arr.2011.03.003
16. Chen H, Goldberg MS, Villeneuve PJ. A systematic review of the relation between long-term exposure to ambient air pollution and chronic diseases. Rev Environ Health. 2008;23(4):243–97.
17. Asogwa OA, Boateng D, Marzà-Florensa A, Peters S, Levitt N, Van Olmen J, et al. Multimorbidity of non-

For peer review only

communicable diseases in low-income and middle-income countries: A systematic review and meta-analysis. BMJ Open. 2022;12(1):1–16.
18. Cezard G, McHale CT, Sullivan F, Bowles JKF, Keenan K. Studying trajectories of multimorbidity: A systematic scoping review of longitudinal approaches and evidence. BMJ Open. 2021;11(11):1–19.
19. Rosbach M, Andersen JS. Patient-experienced burden of treatment in patients with multimorbidity – A systematic review of qualitative data. PLoS One. 2017;12(6):1–18.
20. Price ML, Surr CA, Gough B, Ashley L. Experiences and support needs of informal caregivers of people with multimorbidity: a scoping literature review. Psychol Heal [Internet]. 2020;35(1):36–69. Available from: https://doi.org/10.1080/08870446.2019.1626125
21. Smith SM, O'Kelly S, O'Dowd T. GPs' and pharmacists' experiences of managing multimorbidity: A "Pandora's box." Br J Gen Pract. 2010;60(576):285–94.
22. Lall D, Engel N, Devadasan N, Horstman K, Criel B. Challenges in primary care for diabetes and hypertension: An observational study of the Kolar district in rural India. BMC Health Serv Res. 2019;19(1):1–11.
23. Yellapa V, Devadasan N, Krumeich A, Pai NP, Vadnais C, Pai M, et al. How patients navigate the diagnostic ecosystem in a fragmented health system: A qualitative study from India. Glob Health Action [Internet]. 2017;10(1). Available from: https://doi.org/10.1080/16549716.2017.1350452
24. Pati S, Pati S, van den Akker M, Schellevis FG, Sahoo KC, Burgers JS. Managing diabetes mellitus with comorbidities in primary healthcare facilities in urban settings: a qualitative study among physicians in Odisha, India. BMC Fam Pract. 2021;22(1):1–17.
25. Kinra S, Radha Krishna K V, Kuper H, Rameshwar Sarma K V, Prabhakaran P, Gupta V, et al. Cohort profile: Andhra Pradesh Children and Parents Study (APCAPS). Int J Epidemiol. 2014 Oct;43(5):1417–24.
26. Hartung C, Lerer A, Anokwa Y, Tseng C, Brunette W, Borriello G. Open Data Kit: Tools to Build Information Services for Developing Regions. In: Proceedings of the 4th ACM/IEEE International Conference on Information and Communication Technologies and Development [Internet]. New York, NY, USA: Association for Computing Machinery; 2010. (ICTD '10). Available from: https://doi.org/10.1145/2369220.2369236
27. Bhan N, Madhira P, Muralidharan A, Kulkarni B, Murthy G, Basu S, et al. Health needs, access to healthcare, and perceptions of ageing in an urbanizing community in India: a qualitative study. BMC Geriatr [Internet]. 2017;17(1):156. Available from: http://bmcgeriatr.biomedcentral.com/articles/10.1186/s12877-017-0544-y
28. Pati S, Hussain MA, Swain S, Salisbury C, Metsemakers JFM, Knottnerus JA, et al. Development and Validation of a Questionnaire to Assess Multimorbidity in Primary Care: An Indian Experience. Biomed Res Int. 2016;2016.
29. Salvi S, Apte K, Madas S, Barne M, Chhowala S, Sethi T, et al. Symptoms and medical conditions in 204 912 patients visiting primary health-care practitioners in India: A 1-day point prevalence study (the POSEIDON study). Lancet Glob Heal [Internet]. 2015;3(12):e776–84. Available from: http://dx.doi.org/10.1016/S2214-109X(15)00152-7
30. Hayter AKM, Jeffery R, Sharma C, Prost A, Kinra S. Community perceptions of health and chronic disease in South Indian rural transitional communities: a qualitative study. Glob Health Action [Internet]. 2015;8:25946. Available from: http://www.pubmedcentral.nih.gov/articlerender.fcgi?artid=4323408&tool=pmcentrez&rendertype=abstract
31. Hurst JR, Agarwal G, Van Boven JFM, Daivadanam M, Gould GS, Wan-Chun Huang E, et al. Critical review of multimorbidity outcome measures suitable for low-income and middle-income country settings: Perspectives from the Global Alliance for Chronic Diseases (GACD) researchers. BMJ Open. 2020;10(9):1–9.
32. Abou-Dakn M, Döhmen C, Wenzel S. Validation of the TONOPORT VI ambulatory blood pressure monitor in adults according to the European Society of Hypertension International Protocol revision 2010. J Hum Hypertens. 2017;31(2):89–92.
33. Takahashi H, Yoshika M, Yokoi T. Validation of three automatic devices for the self-measurement of blood pressure according to the European Society of Hypertension International Protocol revision 2010: the Omron HEM-7130, HEM-7320F, and HEM-7500F. Blood Press Monit. 2015;20(2):92–7.
34. Unger T, Borghi C, Charchar F, Khan NA, Poulter NR, Prabhakaran D, et al. 2020 International Society of Hypertension Global Hypertension Practice Guidelines. Hypertension. 2020;75(6):1334–57.
35. Wang TJ, Evans JC, Benjamin EJ, Levy D, LeRoy EC, Vasan RS. Natural history of asymptomatic left ventricular systolic dysfunction in the community. Circulation. 2003;108(8):977–82.

For peer review only

36. Rose GA. The diagnosis of ischaemic heart pain and intermittent claudication in field surveys. Bull World Health Organ. 1962;27:645–58.
37. Gourie-Devi M, Gururaj G, Satishchandra P, Subbakrishna DK. Neuro-Epidemiological Pilot Survey of an Urban Population in a Developing Country. Neuroepidemiology [Internet]. 1996;15(6):313–20. Available from: https://www.karger.com/DOI/10.1159/000109921
38. ElSayed NA, Aleppo G, Aroda VR, Bannuru RR, Brown FM, Bruemmer D, et al. 2. Classification and Diagnosis of Diabetes: Standards of Care in Diabetes-2023. Diabetes Care. 2023;46(1):S19–40.
39. Bril V, Tomioka S, Buchanan RA, Perkins BA. Reliability and validity of the modified Toronto Clinical Neuropathy Score in diabetic sensorimotor polyneuropathy. Diabet Med. 2009;26(3):240–6.
40. van Doremalen RFM, van Netten JJ, van Baal JG, Vollenbroek-Hutten MMR, van der Heijden F. Validation of low-cost smartphone-based thermal camera for diabetic foot assessment. Diabetes Res Clin Pract [Internet]. 2019;149:132–9. Available from: https://doi.org/10.1016/j.diabres.2019.01.032
41. Herraiz-Adillo Á, Cavero-Redondo I, Álvarez-Bueno C, Pozuelo-Carrascosa DP, Solera-Martínez M. The accuracy of toe brachial index and ankle brachial index in the diagnosis of lower limb peripheral arterial disease: A systematic review and meta-analysis. Atherosclerosis. 2020;315(October):81–92.
42. Bedard C, Liu S, Patterson C, Gerstein H, Griffith L. Systematic review: Can non-mydriatic cameras accurately detect diabetic retinopathy? Diabetes Res Clin Pract [Internet]. 2017;129:154–9. Available from: http://dx.doi.org/10.1016/j.diabres.2017.04.024
43. Arokiasamy P, Parasuraman S, Sekher S V, Lhungdim H. Study on global AGEing and adult health (SAGE) Wave 1India National Report. World Health Organisation; 2013.
44. Hanania NA, Mannino DM, Yawn BP, Mapel DW, Martinez FJ, Donohue JF, et al. Predicting risk of airflow obstruction in primary care: Validation of the lung function questionnaire (LFQ). Respir Med [Internet]. 2010;104(8):1160–70. Available from: http://dx.doi.org/10.1016/j.rmed.2010.02.009
45. Sunyer J, Basagana X, Burney P, Anto JM. International assessment of the internal consistency of respiratory symptoms. Am J Respir Crit Care Med. 2000;162(3 I):930–5.
46. Ranzani OT, Bhogadi S, Milà C, Kulkarni B, Balakrishnan K, Sambandam S, et al. Association of ambient and household air pollution with lung function in young adults in an peri-urban area of South-India: A cross-sectional study. Environ Int. 2022;165(May).
47. Chen LK, Woo J, Assantachai P, Auyeung TW, Chou MY, Iijima K, et al. Asian Working Group for Sarcopenia: 2019 Consensus Update on Sarcopenia Diagnosis and Treatment. J Am Med Dir Assoc [Internet]. 2020;21(3):300-307.e2. Available from: https://doi.org/10.1016/j.jamda.2019.12.012
48. Roberts HC, Denison HJ, Martin HJ, Patel HP, Syddall H, Cooper C, et al. A review of the measurement of grip strength in clinical and epidemiological studies: Towards a standardised approach. Age Ageing. 2011;40(4):423–9.
49. Moussavi S, Chatterji S, Verdes E, Tandon A, Patel V, Ustun B. Depression, chronic diseases, and decrements in health: results from the World Health Surveys. Lancet. 2007;370(9590):851–8.
50. Neufeld LM, Larson LM, Kurpad A, Mburu S, Martorell R, Brown KH. Hemoglobin concentration and anemia diagnosis in venous and capillary blood: biological basis and policy implications. Ann N Y Acad Sci. 2019;1450(1):172–89.
51. De Man J, Absetz P, Sathish T, Desloge A, Haregu T, Oldenburg B, et al. Are the PHQ-9 and GAD-7 Suitable for Use in India? A Psychometric Analysis. Front Psychol. 2021;12(May):1–14.
52. Endsley P, Weobong B, Nadkarni A. Psychometric properties of the AUDIT among men in Goa, India. Asian J Psychiatr [Internet]. 2017;29(2017):54–8. Available from: https://doi.org/10.1016/j.ajp.2017.03.006
53. Prince M, Acosta D, Ferri CP, Guerra M, Huang Y, Jacob KS, et al. A brief dementia screener suitable for use by non-specialists in resource poor settings-the cross-cultural derivation and validation of the brief Community Screening Instrument for Dementia. Int J Geriatr Psychiatry. 2011;26(9):899–907.
54. Ministry of Health and Family Welfare (Government of India). Chapter 3-Case finding & diagnosis strategy [Internet]. Technical and Operational Guidelines for TB Control in India. 2011. Available from: https://tbcindia.gov.in/showfile.php?lid=3216
55. World Health Organization. Tuberculosis prevalence surveys: A handbook [Internet]. 2011. Available from: https://apps.who.int/iris/handle/10665/44481
56. Chadha VK, Anjinappa SM, Dave P, Rade K, Baskaran D, Narang P, et al. Sub-national TB prevalence surveys in India, 2006-2012: Results of uniformly conducted data analysis. PLoS One. 2019;14(2):2006–12.
57. World Health Organization. WHO Covid-19: Case Definitions [Internet]. 2020. Available from: WHO/2019-nCoV/Surveillance_Case_Definition/2020.2

For peer review only

58. World Health Organization. A clinical case definition of post COVID-19 condition by a Delphi consensus [Internet]. 2021. Available from: WHO/2019-nCoV/Post_COVID-19_condition/Clinical_case_definition/2021.1
59. Zulewski H, Müller B, Exer P, Miserez AR, Staub JJ. Estimation of tissue hypothyroidism by a new clinical score: Evaluation of patients with various grades of hypothyroidism and controls. J Clin Endocrinol Metab. 1997;82(3):771–6.
60. World Health Organization. Goitre as a determinant of the prevalence and severity of iodine deficiency disorders in populations. 2014.
61. Bhatia SJ, Reddy DN, Ghoshal UC, Jayanthi V, Abraham P, Choudhuri G, et al. Epidemiology and symptom profile of gastroesophageal reflux in the Indian population: Report of the Indian Society of Gastroenterology Task Force. Indian J Gastroenterol. 2011;30(3):118–27.
62. International Institute for Population Sciences (IIPS). Longitudinal Ageing Study in India (LASI) Wave 1, 2017-18, India Report. Mumbai; 2020.
63. UK Biobank. UK Biobank Pain web questionnaire v2.1 [Internet]. 2022 [cited 2023 Mar 14]. Available from: https://biobank.ctsu.ox.ac.uk/crystal/ukb/docs/pain_questionnaire.pdf
64. Aziz Q, Barke A, Bennett MI, Benoliel R, Cohen M, Evers S, et al. A classification of chronic pain for ICD-11. Pain. 2015;156(6):1003–7.
65. Fried LP, Tangen CM, Walston J, Newman AB, Hirsch C, Gottdiener J, et al. Frailty in Older Adults: Evidence for a Phenotype. Journals Gerontol Ser A Biol Sci Med Sci. 2001;56(3):M146–57.
66. Matsuzaki M, Sullivan R, Ekelund U, Krishna KR, Kulkarni B, Collier T, et al. Development and evaluation of the Andhra Pradesh Children and Parent Study Physical Activity Questionnaire (APCAPS-PAQ): A cross-sectional study. BMC Public Health [Internet]. 2016;16(1):1–9. Available from: http://dx.doi.org/10.1186/s12889-016-2706-9
67. Lee EH. Review of the psychometric evidence of the perceived stress scale. Asian Nurs Res (Korean Soc Nurs Sci) [Internet]. 2012;6(4):121–7. Available from: http://dx.doi.org/10.1016/j.anr.2012.08.004
68. Groce NE, Mont D. Counting disability: emerging consensus on the Washington Group questionnaire. Lancet Glob Heal [Internet]. 2017;5(7):e649–50. Available from: http://dx.doi.org/10.1016/S2214-109X(17)30207-3
69. Mactaggart I, Kuper H, Murthy GVS, Oye J, Polack S. Measuring disability in population based surveys: The interrelationship between clinical impairments and reported functional limitations in Cameroon and India. PLoS One. 2016;11(10):1–18.
70. Irace AL, Sharma RK, Reed NS, Golub JS. Smartphone-Based Applications to Detect Hearing Loss: A Review of Current Technology. J Am Geriatr Soc. 2021;69(2):307–16.
71. Renda L, Selçuk ÖT, Eyigör H, Osma Ü, Yılmaz MD. Smartphone based audiometric test for confirming the level of hearing; is it useable in underserved areas? J Int Adv Otol. 2016;12(1):61–6.
72. Aremu S. Evaluation of the hearing test pro application as a screening tool for hearing loss assessment. Niger Med J. 2018;59(5):55.
73. Bright T, Mactaggart I, Kuper H, Murthy G V., Polack S. Prevalence of Hearing Impairment in Mahabubnagar District, Telangana State, India. Ear Hear. 2019;40(1):204–12.
74. World Health Organization. Verbal autopsy standards: ascertaining and attributing causes of death tool [Internet]. 2022 [cited 2023 Mar 14]. Available from: https://www.who.int/standards/classifications/other-classifications/verbal-autopsy-standards-ascertaining-and-attributing-causes-of-death-tool
75. Coates, J., Swindale, A., Bilinsky P. Household Food Insecurity Access Scale (HFIAS) for Measurement of Household Food Access: Indicator Guide (v.3). Washington, D.C.; 2007.
76. Bowen L, Venkatsubbareddy Bharathi A, Kinra S, DeStavola B, Ness A, Ebrahim SD. Development and evaluation of a semi-quantitative food frequency questionnaire for use in urban and rural India. Asia Pac J Clin Nutr. 2012;21(3):355–60.
77. ECRHS. European Community Respiratory Health Survey: Questionnaires and protocols [Internet]. 2018 [cited 2023 Jan 5]. Available from: https://www.ecrhs.org/questionnaires-and-protocols
78. Kroenke K, Spitzer RL, Williams JBW. The PHQ-9: Validity of a brief depression severity measure. J Gen Intern Med. 2001;16(9):606–13.
79. Spitzer RL, Kroenke K, Williams JBW, Löwe B. A brief measure for assessing generalized anxiety disorder: The GAD-7. Arch Intern Med. 2006;166(10):1092–7.
80. World Health Organization. AUDIT: The alcohol use disorders identification test: Guidelines for use in primary health care. World Health Organization; 2001.
81. Cohen S, Williamson G. Perceived stress in a probability sample of the United States [Internet]. Vol. 13,

For peer review only

The Social Psychology of Health. 1988. p. 31–67. Available from: http://doi.apa.org/psycinfo/1988-98838-002
82. Washington Group Short Set on Functioning (WG-SS) [Internet]. 2022. Available from: https://www.washingtongroup-disability.com/question-sets/wg-short-set-on-functioning-wg-ss/
83. Jyani G, Sharma A, Prinja S, Kar SS, Trivedi M, Patro BK, et al. Development of an EQ-5D Value Set for India Using an Extended Design (DEVINE) Study: The Indian 5-Level Version EQ-5D Value Set. Value Heal [Internet]. 2022; Available from: https://doi.org/10.1016/j.jval.2021.11.1370
84. Lamb SE, Jørstad-Stein EC, Hauer K, Becker C. Development of a common outcome data set for fall injury prevention trials: The Prevention of Falls Network Europe consensus. J Am Geriatr Soc. 2005;53(9):1618–22.
85. World Health Organization. Verbal autopsy standards: ascertaining and attributing causes of death tool. 2022.
86. HearingTest app [Internet]. Available from: https://www.e-audiologia.pl/HearingTest/
87. Sorensen TB, Wilson R, Gregson J, Shankar B, Dangour AD, Kinra S. Is night-time light intensity associated with cardiovascular disease risk factors among adults in early-stage urbanisation in South India? A cross-sectional study of the Andhra Pradesh Children and Parents Study. BMJ Open. 2020;10(11):1–11.
88. Sanchez M, Ambros A, Milà C, Salmon M, Balakrishnan K, Sambandam S, et al. Development of land-use regression models for fine particles and black carbon in peri-urban South India. Sci Total Environ [Internet]. 2018;634:77–86. Available from: https://doi.org/10.1016/j.scitotenv.2018.03.308
89. Shi D, Lin Z, Wang W, Tan Z, Shang X, Zhang X, et al. A Deep Learning System for Fully Automated Retinal Vessel Measurement in High Throughput Image Analysis. Front Cardiovasc Med. 2022;9(March):1–12.
90. Ng SK, Tawiah R, Sawyer M, Scuffham P. Patterns of multimorbid health conditions: A systematic review of analytical methods and comparison analysis. Int J Epidemiol. 2018;47(5):1687–704.
91. May CR, Eton DT, Boehmer K, Gallacher K, Hunt K, MacDonald S, et al. Rethinking the patient: using Burden of Treatment Theory to understand the changing dynamics of illness. BMC Health Serv Res. 2014;14:281.
92. Pati S, MacRae C, Henderson D, Weller D, Guthrie B, Mercer S. Defining and measuring complex multimorbidity: a critical analysis. Br J Gen Pract [Internet]. 2023 Aug 1;73(733):373 LP – 376. Available from: http://bjgp.org/content/73/733/373.abstract
93. Braun V, Clarke V. Can I use TA? Should I use TA? Should I not use TA? Comparing reflexive thematic analysis and other pattern-based qualitative analytic approaches. Couns Psychother Res. 2021;21(1):37–47.

Author statement

JL, SKu, PM, HM, OK, SS, BK and SK were involved in conception of the study. JL, SKu, PM, HM, SB, SA, NB, WS, AS, OK, GI, SKK, RK, SS, TD, BK and SK were involved in design and development of the study protocol. JL and SKu contributed equally to developing the manuscript. All authors substantially contributed and commented on the manuscript and approved the final version.

Conflicts of interest

None.

Funding

This work was primarily supported by the UK Medical Research Council (MRC) grant number MC_PC_MR/T038292/1. This research was also part-funded by MRC grant number MR/V001221/1, the Nagasaki University "Doctoral Program for World-leading Innovative and Smart Education" for Global Health, KENKYU SHIDO KEIHI, and in-kind support from ICMR-NIN.

Acknowledgments

For peer review only

We would like to acknowledge and thank the various researchers and practitioners in each partner institution and Ranga Reddy healthcare system that advised on elements of the study protocol, village leaders and local stakeholders for supporting and facilitating data collection, ICMR-NIN for permitting use of their facilities for data collection, the hard-working field team, and most imperatively, the participants who kindly gave us their time.

Figure legend

Figure 1: Overview of feasible analyses examining the determinants, burden and consequences of multimorbidity using previously collected (2010-12) and planned (2022-23) APCAPS data.

*** Available in 2016. ^ Mortality data will be collected prospectively.**

For peer review only

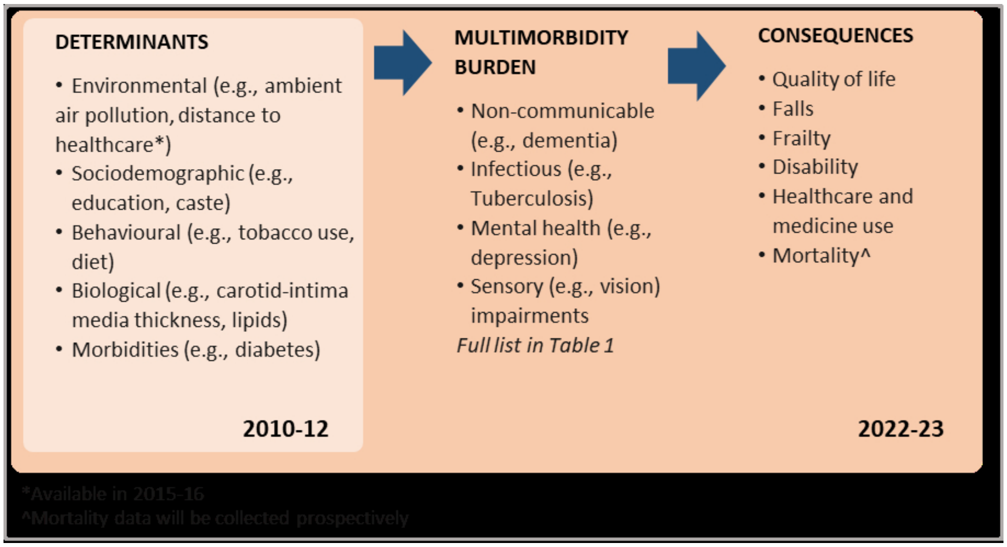

Figure 1:Overview of feasible analyses examining the determinants, burden and consequences of multimorbidity using previously collected (2010-12) and planned (2022-23) APCAPS data.* Available in 2016. ^ Mortality data will be collected prospectively.

392x211mm (59 x 59 DPI)
